# A comparative genomic analysis of putative pathogenicity genes in the host-specific sibling species *Colletotrichum graminicola* and *Colletotrichum sublineola*

**DOI:** 10.1186/s12864-016-3457-9

**Published:** 2017-01-10

**Authors:** E. A. S. Buiate, K. V. Xavier, N. Moore, M. F. Torres, M. L. Farman, C. L. Schardl, L. J. Vaillancourt

**Affiliations:** 1 Department of Plant Pathology, University of Kentucky, 201F Plant Science Building, 1405 Veterans Drive, Lexington, KY 40546-0312 USA; 2 Present Address: Monsanto Company Brazil, Uberlândia, Minas Gerais Brazil; 3 Department of Computer Science, University of Kentucky, Davis Marksbury Building, 328 Rose Street, Lexington, KY 40504-0633 USA; 4 Present Address: Functional Genomics Laboratory, Weill Cornell Medicine, Doha, Qatar

**Keywords:** Fungal virulence, Maize anthracnose, Sorghum anthracnose, Fungal secondary metabolism, Fungal effectors, Hypersensitive response, Effector-triggered immunity, Plant disease

## Abstract

**Background:**

*Colletotrichum graminicola* and *C. sublineola* cause anthracnose leaf and stalk diseases of maize and sorghum, respectively. In spite of their close evolutionary relationship, the two species are completely host-specific. Host specificity is often attributed to pathogen virulence factors, including specialized secondary metabolites (SSM), and small-secreted protein (SSP) effectors. Genes relevant to these categories were manually annotated in two co-occurring, contemporaneous strains of *C. graminicola* and *C. sublineola*. A comparative genomic and phylogenetic analysis was performed to address the evolutionary relationships among these and other divergent gene families in the two strains.

**Results:**

Inoculation of maize with *C. sublineola*, or of sorghum with *C. graminicola*, resulted in rapid plant cell death at, or just after, the point of penetration. The two fungal genomes were very similar. More than 50% of the assemblies could be directly aligned, and more than 80% of the gene models were syntenous. More than 90% of the predicted proteins had orthologs in both species. Genes lacking orthologs in the other species (non-conserved genes) included many predicted to encode SSM-associated proteins and SSPs. Other common groups of non-conserved proteins included transporters, transcription factors, and CAZymes. Only 32 SSP genes appeared to be specific to *C. graminicola*, and 21 to *C. sublineola*. None of the SSM-associated genes were lineage-specific. Two different strains of *C. graminicola*, and three strains of *C. sublineola,* differed in no more than 1% percent of gene sequences from one another.

**Conclusions:**

Efficient non-host recognition of *C. sublineola* by maize, and of *C. graminicola* by sorghum, was observed in epidermal cells as a rapid deployment of visible resistance responses and plant cell death. Numerous non-conserved SSP and SSM-associated predicted proteins that could play a role in this non-host recognition were identified. Additional categories of genes that were also highly divergent suggested an important role for co-evolutionary adaptation to specific host environmental factors, in addition to aspects of initial recognition, in host specificity. This work provides a foundation for future functional studies aimed at clarifying the roles of these proteins, and the possibility of manipulating them to improve management of these two economically important diseases.

**Electronic supplementary material:**

The online version of this article (doi:10.1186/s12864-016-3457-9) contains supplementary material, which is available to authorized users.

## Background

Members of the fungal genus *Colletotrichum* cause anthracnose diseases on nearly every plant species grown for food or fiber worldwide [1, 2]. *Colletotrichum graminicola* (Ces.) Wils., and *C. sublineola* Henn., cause economically important anthracnose leaf blight and stalk rot diseases of maize (*Zea mays* L.), and sorghum (*Sorghum bicolor* [L.] Moench), respectively [3–6]. These two fungal sibling species are morphologically very similar, but reproductively isolated [5]. Results of molecular phylogenetic analyses suggest that they diverged from a common ancestor relatively recently, perhaps at the same time as the split between maize and sorghum (thought to be approximately 12 million years ago) [4, 5, 7–11]. There are no reports in the literature of *C. graminicola* infecting sorghum or of *C. sublineola* infecting maize in the field, and most studies agree that the two species are host-specific [6, 12–14]. We have found that *C. sublineola* can infect maize stalk epidermal cells, and maize leaf sheath cells that are dead or dying [15, 16]. This ability of *C. sublineola* to conditionally infect some maize tissues might explain two earlier papers that reported that maize was susceptible to isolates of *Colletotrichum* from sorghum [17, 18]. It also suggests that host range is determined by active recognition of and response to the non-pathogen by healthy tissues of the non-host, rather than structural barriers or the absence of some vital nutrient or other factor.

The determination of host range in plant pathogens is often attributed to the presence or absence of pathogen virulence factors, particularly specialized secondary metabolites (SSMs), and small-secreted protein (SSP) effectors [19–25].

The presence of particular SSMs has been associated with the determination of host range in some phytopathogenic fungi including *Alternaria* spp. [21] and *Cochliobolus* spp. [20]. The major classes of fungal SSMs include polyketides, peptides, terpenes, and indole alkaloids [26–28]. Each of these classes is associated with a specific family of proteins. These SSM-associated proteins are: polyketide synthases (PKS); nonribosomal peptide synthetases (NRPS); terpene synthases (TS); and dimethylallyl transferases (DMAT), respectively. Genes encoding these enzymes and other proteins involved in the production of the SSMs are often found physically associated in transcriptionally co-regulated gene clusters [29, 30].

Fungal effectors have been defined as SSPs that alter the structure or modulate the function of host cells to facilitate infection [31, 32]. Some effectors are translocated and operate in the host cytoplasm [33–36]. Others function in the plant cell apoplast [37]. Some effectors act as host specific toxins and induce apoptosis only in certain plant genotypes, conferring host specificity in several important necrotrophic pathogens [38, 39]. Examples of known effector categories include serine proteases, necrosis and ethylene-inducing protein 1-like proteins (NEP1-like proteins), and small cysteine-rich proteins [23, 40, 41].

Some plants have evolved an ability to recognize and respond to certain effectors by activating defense pathways via specific resistance (R) proteins, a phenomenon known as effector-triggered immunity (ETI). In these cases, the effectors act as avirulence (Avr) factors. Multiple rounds of mutation and selection of R and Avr genes during a co-evolutionary “arms-race” leads to the presence of multiple pathogenic races expressing different combinations of Avr genes within the pathogen population [42]. Recent evidence suggests that inducible non-host resistance in many agriculturally-important pathosystems, particularly involving closely related hosts, is due to ETI. In these cases all members of the non-host plant species contain the same R gene(s), whereas all members of the nonpathogenic microbial species contain the corresponding Avr gene(s) [43–52].

A number of recent comparative genomics studies have confirmed that genes encoding SSM-associated proteins and SSPs show evidence of rapid evolution in related pathogens with different host ranges [20, 25, 53–65]. Most of these studies have involved comparisons of relatively distantly related pathogens, and/or strains with diverse geographic origins. There have been comparatively few analyses of co-occurring, closely related sibling species. The goal of the present work was to identify, characterize, and compare candidate host specificity-related genes from two contemporaneous, co-occurring, host-specific strains of the sibling species *C. graminicola* and *C. sublineola*.

## Results and discussion

### The cytology of host specificity

**Fig. 1:**
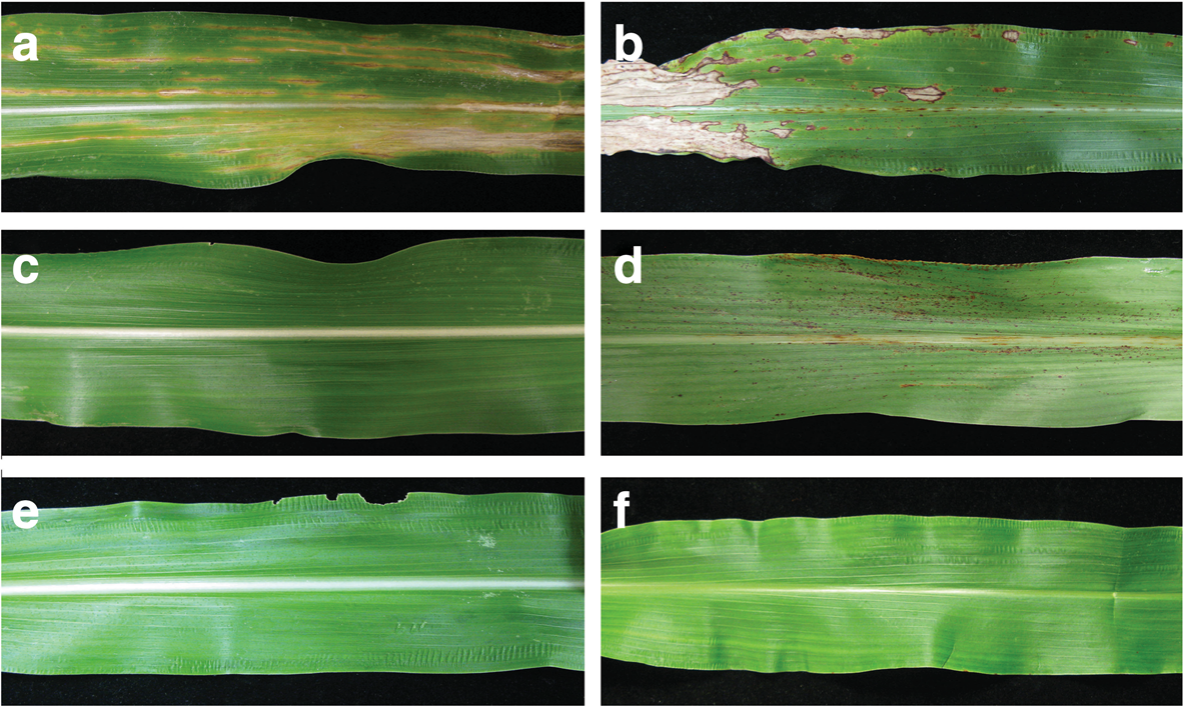
**a** maize leaf inoculated with *C. graminicola*, 7 dpi; **b** sorghum inoculated with *C. sublineola*, 7 dpi; **c** maize inoculated with *C. sublineola*, 7 dpi; **d** sorghum inoculated with *C. graminicola*, 7 dpi; **e** maize control, mock-inoculated with water, 7 dpi; **f** sorghum control, mock-inoculated with water, 7 dpi

**Fig. 2:**
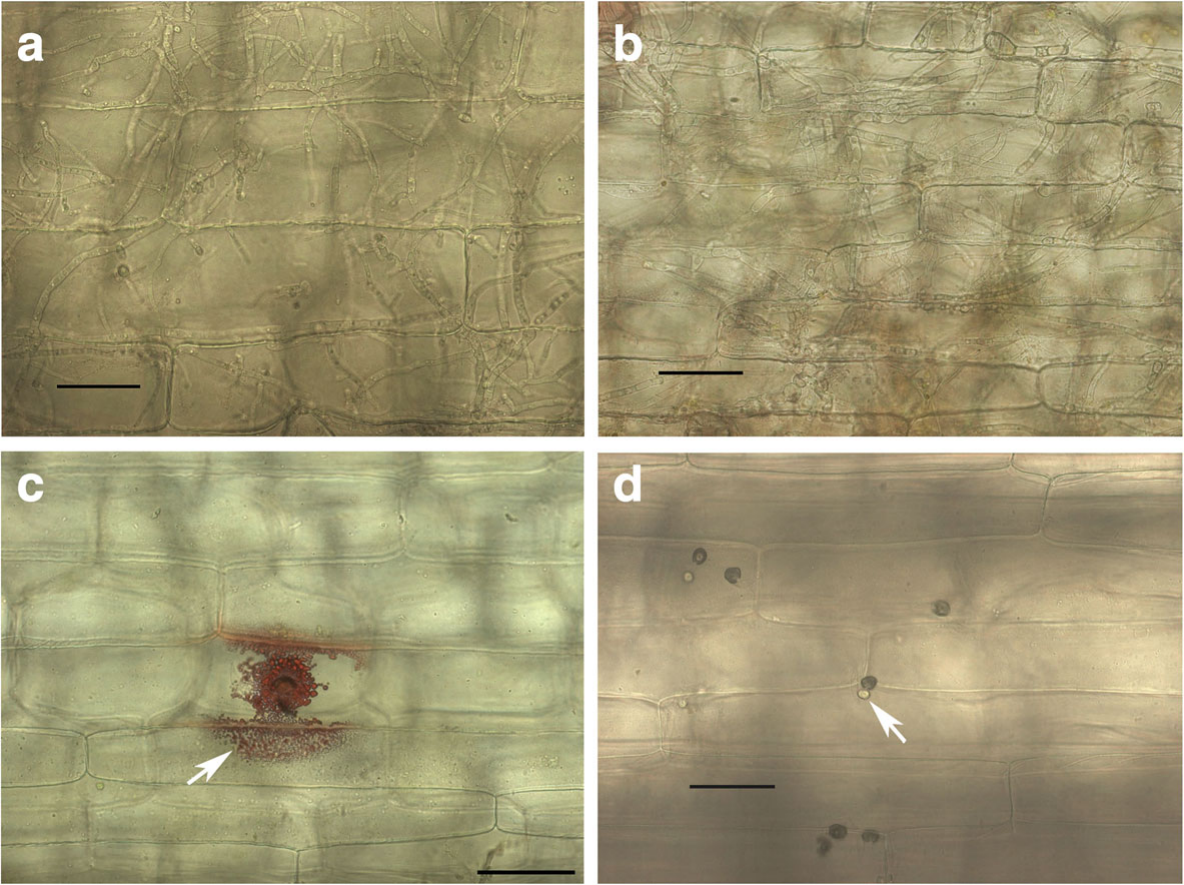
**a** *C. graminicola* hyphae in maize leaves, 48 hpi; **b** *C. sublineola* hyphae in sorghum leaves, 72 hpi; **c** *C. graminicola* on sorghum, 48 hpi, *white arrow* indicates red vesicles surrounding the appressorium; **d** *C. sublineola* on maize, 48 hpi, *white arrow* indicates an iridescent papillum beneath a melanized appressorium. Scale bars equal to 50 μm

**Fig. 3:**
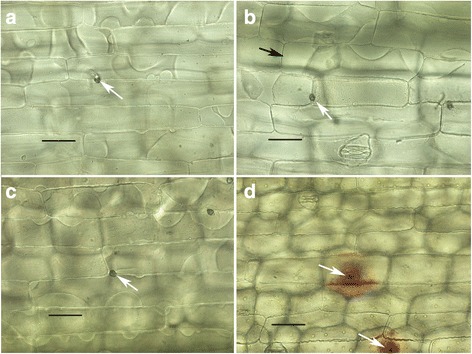
**a** CgSl1 on maize sheath, 48 hpi. Cell beneath appressorium (*white arrow*) plasmolyzes normally; **b** CgSl1 on maize sheath, small penetration hypha (*white arrow*) 48 hpi. Adjacent cell (*black arrow*) plasmolyzes normally. Cell containing penetration hyphae appears granulated, plasma membrane visible but appears abnormal; **c** M1.001 on sorghum sheath, 24 hpi, cells beneath appressoria (*white arrow*) still plasmolyze; **d** M1.001 on sorghum sheath, 48 hpi. No plasmolysis evident in any of the cells in the vicinity of the appressoria (*white arrow*). Scale bars equal to 50 μm

**Fig. 4:**
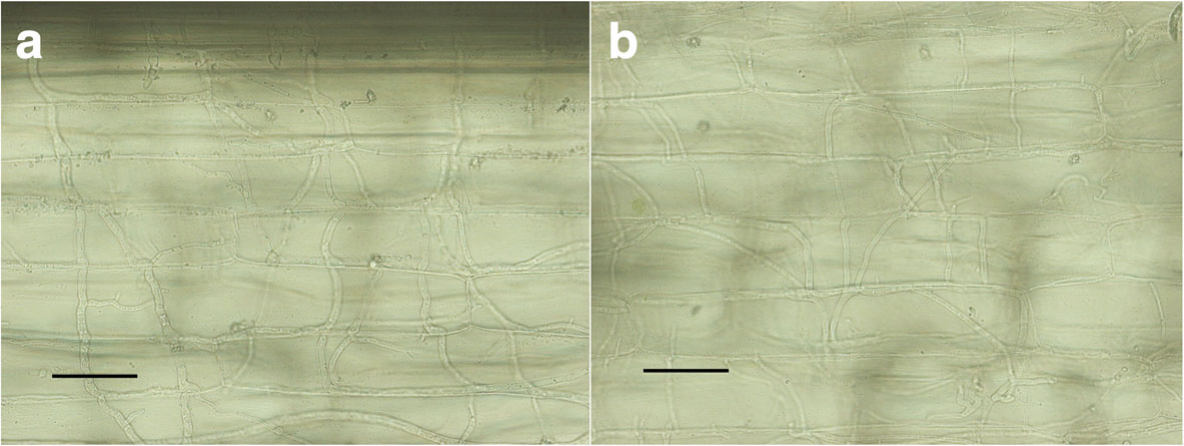
**a** CgSl1 growing in cells of maize sheaths killed by liquid nitrogen, 48 hpi; **b** M1.001 growing in cells of sorghum sheaths killed by liquid nitrogen, 48 hpi. Scale bars equal to 50 μm

*Colletotrichum graminicola* strain M1.001 was isolated from maize in Missouri in the late 1970s [66]. This strain caused typical, sporulating anthracnose lesions on maize leaves (cv. Mo17) within 3 days post inoculation (dpi), but on leaves of sorghum (cv. Sugar Drip) it produced only small reddish flecks, which failed to expand or sporulate even up to 7 dpi (Fig. 1a, d). *Colletotrichum sublineola* strain CgSl1 was isolated in the early 1980s from grain sorghum in Indiana [6]. This strain caused large, sporulating anthracnose lesions on sorghum, but not on maize leaves (Fig. 1b, c). *Colletotrichum graminicola* strain M1.001 readily infected and colonized multiple cells of detached leaf sheaths of maize by 48 h after inoculation (hpi) and *C. sublineola* strain CgSl1 did the same in sorghum sheaths by 72 hpi (Fig. 2a, b). In contrast, *C. graminicola* failed to infect leaf sheath cells of sorghum, and *C. sublineola* failed to infect maize leaf sheath cells, even up to 6 dpi (Fig. 2c, d). Sorghum responded within 48 hpi to *C. graminicola* appressoria by an accumulation of numerous vesicles containing red pigments, and maize responded to *C. sublineola* appressoria by the formation of iridescent papillae (Fig. 2c, d). Previous studies have determined that the red pigments consist of various anthocyanidin phytoalexins [67]. The maize papillae are composed primarily of callose [68]. Visible primary hyphae were always very small, and were produced in fewer than 1% of infection attempts in both non-host combinations. Unpenetrated cells beneath *C. sublineola* appressoria in maize leaf sheaths typically retained their ability to plasmolyze even up to 48 hpi, but cells containing rare penetration hyphae appeared granulated, and did not plasmolyze normally (Fig. 3a, b). Sorghum cells beneath *C. graminicola* appressoria usually plasmolyzed at 24 hpi, but by 48 hpi most of the cells had lost the ability to plasmolyze, whether they contained infection hyphae or not (Fig. 3c, d, Additional file 1: Figure S1). Most of the cells in the mock-inoculated maize and sorghum controls still plasmolyzed normally up to 72 hpi (Additional file 2: Figure S2). *Colletotrichum sublineola* and *C. graminicola* were able to colonize non-host leaf sheaths readily if the cells were killed first by a localized application of liquid nitrogen (Fig. 4a, b). These observations suggest that host specificity is based on active recognition of the non-pathogen by living non-host plant cells, followed by rapid deployment of defense responses targeting the infection sites, and ultimately plant cell death prior to, or coincident with, penetration. To identify potential candidates for factors that might trigger or facilitate this recognition, we compared the genomes of these two strains, with a particular focus on genes that were not conserved between them, and on genes encoding putative SSPs and SSM-associated proteins.

### The genomes of the *C. graminicola* and *C. sublineola* strains are very similar to one another, confirming their close evolutionary relationship

*Colletotrichum graminicola* and *C. sublineola* belong to a monophyletic clade of closely related *Colletotrichum* fungi that affect various graminaceous hosts [9, 10, 69]. We sequenced, assembled, and analyzed the genome of the CgSl1 strain of *C. sublineola,* and compared it with the previously published genome assembly and annotation of *C. graminicola* strain M1.001 [69]. The *C. sublineola* assembly was approximately 20% larger than the published M1.001 genome assembly (Table 1), although the amount of single-copy DNA was similar (Table 2). The *C. sublineola* genome was predicted to encode about 1300 more genes than the number previously published for *C. graminicola* [69] (Table 1, Additional file 3). Both genome annotations contained homologs for most or all of a set of 248 phylogenetically conserved genes, as identified by CEGMA, aka. the Core Eukaryotic Genes Mapping Approach [70], suggesting that both are relatively complete (Table 1).

**Table 1:** Characteristics of the genome assemblies that were used in this study

Genome features annotation	*C. graminicola M1.001 BROAD*	*C. graminicola M5.001 MAKER*	*C. sublineolum CgSl1 MAKER*
Source of Strain, Reference	Maize, Missouri, 1978 [66]	Maize, Brazil, 1988 [119]	Sorghum, Indiana, 1982 [6]
NCBI accession no. of assembly	ACOD01000001	MRBI01000001	MQVQ01000001
Reference	[69]	This Manuscript	This Manuscript
Strain	M1.001	M5.001	CgSl1
Total contig length (Mb)	51.6	48.8	64.8
Number of Contigs	1,151	3,280	12,943
Number of scaffolds	653	n/a^a^	548
N_50_ contig (kb)	228.96	69.85	41.05
N_50_ scaffold (Kb)	579.19	n/a^a^	339.27
GC-content (%)	49.12	49.8	46.45
Protein-coding genes	12,006	15,052	13,311
Conserved Genes [71]	248	248	247
Mean transcript length (bp)	1399.1	1224.9	1389.9
Number of exons	32967	64326	35389
Mean number of introns/gene	1.745	3.2126	1.65
Mean Intergenic distance (bp)	2691	1599	2224.7
Percentage coding	32.81	36.89	29.5
Percentage repetitive DNA in genome assembly	3.12	2.83	3.14

^a^ This assembly was not scaffolded

**Table 2:** Results of a blastn analysis of genome similarity among three species of *Colletotrichum*

	*C. graminicola* (M1.001)	*C. sublineola* (CgSl1)	*C. higginsianum*
Total Genome Assembly Size (N’s removed)	50,866,947	63,193,777	49,075,584
Single Copy Portion	42,075,489	46,160,404	42,288,585
Total Alignable Single-Copy DNA: *C. graminicola*	NA	23,584,235	9,885,330
Total Alignable Single-Copy DNA: *C. sublineola*	23,584,235	NA	9,919,326
SNPS per 1 Mb alignable, single-copy DNA: *C. graminicola*	NA	100,146	127,026
SNPS per 1 Mb alignable, single-copy DNA: *C. sublineola*	100,146	NA	129,126

Partial sequences of four genes have been used previously for multigene phylogenetic analysis of *Colletotrichum* [69]. These included portions of the *ACT* gene; the *CHS* gene; the *HIS3* gene; and the *TUB2* gene. These sequences from CgSl1 shared 100% identity with those of strain S.3001, the designated epitype specimen for *C. sublineola* [10, 69] (Additional file 4: Figure S3). The internal transcribed spacer (ITS) sequence from CgSl1 also shared 99.6% identity with the ITS sequence of S3.001 [10]. This confirms that CgSl1 belongs to the *C. sublineola* species as it is presently defined (Additional file 4: Figure S3).

Approximately 50% of the single-copy DNA sequence in the CgSl1 and M1.001 assemblies could be directly aligned by blastn (Table 2). In comparison, only about 23% of the assembly of *C. higginsianum*, a more distantly related species pathogenic on Brassicaceae, and belonging to a sister clade [69, 71], could be aligned with either of these two genomes (Table 2). As expected, there were also fewer single nucleotide polymorphisms (SNPs) per Mb of alignable single-copy DNA between *C. graminicola* and *C. sublineola* than between *C. higginsianum* and the other two genomes (Table 2).

**Fig. 5:**
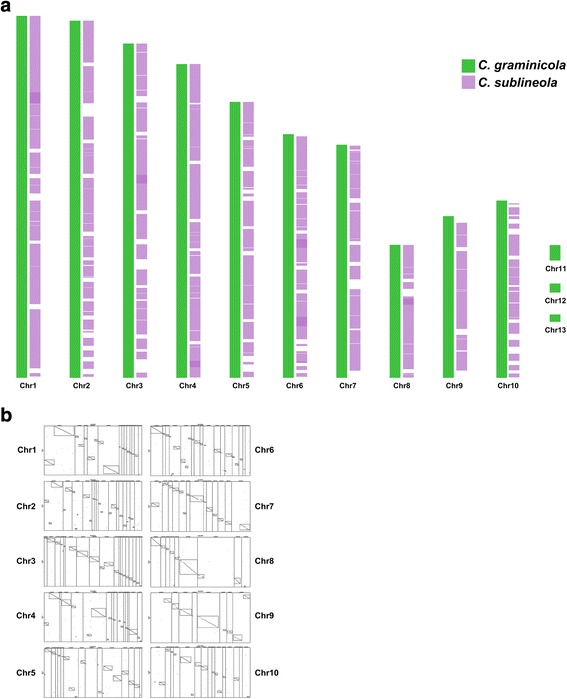
**a** *C. sublineola* scaffolds anchored to *C. graminicola* chromosomes (chromosome optical map of *C. graminicola* published in [69]. **b** Microsynteny between *C. sublineola* contigs and *C. graminicola* chromosomes. Each panel illustrates a different chromosome. The three *C. graminicola* minichromosomes are not included in the figure

Eighty-three percent of the *C. graminicola* genome assembly could be aligned with *C. sublineola* scaffolds based on the relative arrangement of conserved genes (Fig. 5a, Table 3). More than 80% of the *C. graminicola* and *C. sublineola* genes were syntenous (Table 3). Regions that appear to be translocated and/or inverted, and small “islands” that appeared to lack synteny, could be discerned embedded within the largely co-linear assemblies (Fig. 5b). No part of the *C. sublineola* assembly could be aligned with the three *C. graminicola* minichromosomes (Fig. 5a), which seem to be unique to this strain of *C. graminicola* [72].

**Table 3:** Genome synteny between *C. graminicola* strain M1.001 and *C. sublineola* strain CgSl1

	Synteny blocks		% Coverage	Mean block length (Kb) ^a^	Number of genes included in synteny blocks	% Genes included in synteny blocks^b^	Mean number of genes per synteny block^c^
*C. graminicola x C. sublineola*	182	*C. graminicola*	83	223	9874	83	54.25
		*C. sublineola*	77	234	9884	86	54.31

^a^ Calculated as SyMap Total Kb * % coverage/ # blocks

^b^ Number of distinct genes that overlap a synteny anchor assigned to a synteny block

^c^ Calculated as # genes hit/ # blocks

### *Colletotrichum graminicola* and *C. sublineola* encode similar proteins and protein families

The Protein Family Database (Pfam) [73] was used to characterize and compare predicted proteins from *C. graminicola* and *C. sublineola* (Additional file 5: Table S1). Only 67% of *C. graminicola* proteins, and 62% of *C. sublineola* proteins, could be categorized into Pfam families. Most of these families were shared by both isolates, with relatively few differences in the number of family members across the strains. There were 13 families in which there was at least a three-fold expansion in one species versus the other (Additional file 5: Table S1). For example, *C. sublineola* appeared to be enriched in some SSM domains, and in one family of phosphotransferase enzymes, in comparison with *C. graminicola*. There were 82 Pfam families that were found only in *C. graminicola*, while 73 were found only in *C. sublineola* (Additional file 5: Table S1). Nearly all of these non-conserved families contained only a single protein, and relatively few (26% for *C. sublineola* and 13% for *C. graminicola*) included members that have been previously implicated in pathogenicity, based on comparisons to the Pathogen-Host Interactions database (PHI-base), which catalogs pathogenicity-associated genes that have been identified in a variety of pathogenic microbes [74, 75] (Additional file 5: Table S1).

### The *C. graminicola* and *C. sublineola* annotations each include more than 1000 predicted proteins that are not shared between the two species

Ortho-MCL [76] was used initially to identify putative orthologous (aka. shared) proteins from *C. graminicola* and *C. sublineola*. Results indicated that *C. graminicola* and *C. sublineola* shared more than 90% of their proteins (Table 4, Additional file 5: Tables S2, S3). They shared fewer proteins with their more distant relative *C. higginsianum*, but all three species still had more than 85% of their proteins in common (Table 4, Additional file 5: Tables S2, S3).

**Table 4:** Summarized data of Ortho-MCL and RBH analysis of predicted proteins from *C. graminicola* and *C. sublineola*

Shared by:	*C. graminicola*		Shared by:	*C. sublineola*	
	OrthoMCL	RBH		OrthoMCL	RBH
CgramCsubChigg	9281	9264	Cgram:Csub:Chigg	9323	9252
Cgram:Csub	1015	1018	Csub:Cgram	1036	1057
Cgram:Chiggs	473	560	Csub:Chiggs	333	500
Cgram only	134	1164	Csub only	456	2502
Not Characterized	1103	0	Not Characterized	2163	0
Total	12006	12006	Total	13311	13311

Approximately 9% of *C. graminicola* predicted proteins, and 16% of *C. sublineola* predicted proteins, were not assigned to ortholog groups by Ortho-MCL (Table 4, Additional file 5: Tables S2, S3). Thus, the Reciprocal BLAST Hits (RBH) approach [77] was also used to identify putative orthologous proteins. With this approach, all proteins could be accounted for. For more than 90% of the proteins, RBH gave the same result as Ortho-MCL (Additional file 5: Tables S2, S3). Because the RBH included all of the predicted proteins, these results were used for subsequent analyses. The results indicated that the *C. graminicola* annotation included 1724 proteins that were not found in *C. sublineola* (Table 4; Additional file 5: Table S2), while the CgSl1 annotation included 3002 proteins that were not shared with M1.001 (Table 4; Additional file 5: Table S3). These proteins will hereafter be referred to as non-conserved proteins (NCPs). Almost one third of the M1.001 NCPs, and 17% of the CgSl1 NCPs, were shared with the more distantly-related *C. higginsianum*, suggesting a role for loss as well as gain of genes in the evolutionary history of these species (Additional file 5: Tables S2, S3).

Mapping of the genes encoding NCPs of *C. graminicola* to the *C. sublineola* genome assembly, and vice versa, revealed that between one third and one half of them (48% in *C. graminicola*, and 30% in *C. sublineola*) matched sequences in the other genome assembly (Additional file 5: Tables S4, S5). These sequences might represent homologs that were not annotated due to assembly fragmentation or to differences in the gene-calling parameters of the two annotation programs. They could also represent mutant alleles (e.g. nonsense mutations) that were not recognized as ORFs. More detailed studies will be necessary to determine which of these possibilities applies to each sequence.

### Characteristics of the *C. graminicola* and *C. sublineola* NCPs

The predicted proteins that were not shared between the two *Colletotrichum* species were relatively small, with an average size of less than 300 aa, compared with an average of more than 460 aa for all proteins (Additional file 5: Tables S4, S5). A majority in each case (60% of *C. graminicola* NCPs, and 70% of *C. sublineola* NCPs) were not classified by Ortho-MCL (Additional file 5: Tables S4, S5). Transcript data for *C. sublineola* are not available, but 50% of the NCPs of *C. graminicola* were supported by transcript evidence *in planta* (Additional file 5: Table S4) [78]. This could indicate that the rest of the predicted *C. graminicola* NCP genes are not really genes. It could also mean that NCP genes tend to be expressed at especially low levels, or under very specific circumstances that were not achieved in our *in planta* transcriptome analysis. Further studies will be necessary to address these different possibilities.

About half of the NCPs in both *C. graminicola* and *C. sublineola* were predicted to localize to either mitochondria or nuclei (Table 5; Additional file 5: Tables S4, S5). Only about 15% in each species were predicted to be secreted, and another 10% were predicted to localize to the plasma membrane.

**Table 5:** Numbers of non-conserved proteins of *C. graminicola* and *C. sublineola* that are predicted to localize to various locations

Predicted Location	*C. graminicola*	*C. sublineola*
Cyto-mito	11	17
Cyto-nucl	62	116
Cyto-pero	2	3
Cytoskeleton	66	94
Cytosol	288	531
Endoplasmic reticulum	3	2
Extracellular	232	447
Mito-nucl	4	4
Mitochondria	449	715
Nuclear	420	827
Peroxisome	4	3
Plasma membrane	183	239
No Prediction	0	4
Total	1724	3002

The high number of predicted nuclear proteins among the NCPs may suggest that there have been shifts in the regulation of gene expression in these two species that have had important impacts on host specificity. Some of these NCPs may also specifically target the host nucleus: for example, one of the predicted nuclear proteins in *C. graminicola* was GLRG_04079, aka. CgEP1, recently characterized as an essential *C. graminicola* effector that is targeted to the plant nucleus, with both a secretion signal and a nuclear localization signal (NLS) [79] (Additional file 5: Table S4). In our study, neither SignalP nor WoLF PSORT indicated the presence of a signal peptide in this protein. A second candidate nuclear effector identified in [79], GLRG_03517, was similarly not predicted to have a signal peptide in our study. A third putative NLS effector from that study (GLRG_08510) was on our list of NCPs as a predicted SSP, but not as a nuclear protein. These differences in predicted locations probably relate to differences in the localization prediction protocols that we used. This illustrates why localization predictions should be experimentally confirmed. The rest of the NLS effectors identified in [79] are conserved in CgSl1, and thus they were not among the NCPs.

Approximately a quarter of the NCPs in each species were predicted to be localized in the mitochondria (Table 5). Mitochondrial proteins have been implicated in several important animal disease mechanisms [80–82]. In animal cells, some transcription factors and receptors are known to translocate to the mitochondria in response to extracellular signals, where they promote cell death or cell survival [83]. The high number of predicted mitochondrial proteins among the *Colletotrichum* NCP may point to an important role for mitochondrial functions in host adaptation and specificity in these two species. However, the locations of these proteins in the mitochondria should be confirmed by more direct methods before drawing any definitive conclusions.

The NCPs were further evaluated by blastx against the NCBI nr database, and also against the predicted proteomes of the *C. sublineola* epitype strain, and of five other closely related species of *Colletotrichum* isolated from gramineaceous hosts [10]. The latter can be accessed from the Joint Genome Institute (JGI) genome portal (http://genome.jgi.doe.gov/). Based on this analysis, about 20% (361/1724) of the NCPs in *C. graminicola*, and about 25% (736/3002) of the *C. sublineola* NCPs, appeared to be lineage-specific (LS). Although the number of LS genes may decrease as new fungal genomes are added to the databases, the lack of homologs in the five closely related species should make this less likely.

A majority (>65%) of the NCPs in both strains did not match any Pfam categories (Table 6). About 10% of these non-classified NCPs in each case were putative SSPs. Among the minority of NCPs with Pfam classifications, the largest groups consisted of transporters; cytochrome P450s; SSM-associated proteins; carbohydrate-active enzymes (CAZymes); and transcription factors (Table 6). There was also a large group of proteins in each case categorized as heterokaryon incompatibility factors, and a number of other proteins that could potentially be involved in signaling (e.g. protein kinases and protein phosphatases), and pathogenicity, e.g. proteins with LysM chitin-binding domains [84]; necrosis-inducing NPP domains [85]; NUDIX domains [86, 87]; and Common in Fungal Extracellular Membrane (CFEM) domains [88]. Seventeen percent of the *C. sublineola* NCPs, and 20% of the *C. graminicola* NCPs, matched entries in the PHI database. The NCPs for each species were comprised of similar classes, but the CgSl1 annotation generally included more members of each class than the M1.001 annotation, accounting for the larger number of NCPs predicted overall in the *C. sublineola* strain (Table 6).

**Table 6:** Numbers of non-conserved proteins in *C. graminicola* and *C. sublineola* in various categories

Protein Category		*C. graminicola*	*C. sublineola*
No Pfam		1123	2180
Pfam category	Transcription Factors	22	25
Pfam category	Glycosyl Hydrolases	19	22
Pfam category	Heterokaryon Incompatibility Proteins	13	24
Pfam category	Transporters (MFS, ABC, and other)	54	55
Pfam category	Cytochrome P450s	23	56
Pfam category	NUDIX domain	3	1
Pfam category	CFEM domain	2	0
Pfam category	Necrosis inducing NPP domain	2	5
Pfam category	Protein kinase domain	3	15
Pfam category	Secondary Metabolism	13	33
SSP Putative Effectors		143	301
Cysteine-rich SSP		64	111
Cazymes Database		62	73
Members of Secondary Metabolism Clusters		78	13
Hits to PHI-base		343	503
Hits to MEROPS Secreted Peptidase Database		14	13
Hits to Transporters Database		127	161

Transporters represented a major category of the NCPs with Pfam designations, and included members of several different superfamilies (Additional file 5: Tables S4, S5). The largest group belonged to the Major Facilitator Superfamily (MFS). MFS transporters are the most common category of secondary carrier proteins. Members of this group are involved in the uptake of essential minerals and nutrients, also serving in many cases as nutrient sensors [89]. Many of the other overrepresented categories of MFS transporters function in the transport of various drugs and toxins [90], and include members that are homologs of known toxin-associated genes in other fungi (Additional file 5: Tables S4, S5). Another well-represented group of NCP transporters, the ATP-Binding Cassette (ABC) Superfamily, are also known to have important functions in the transport of toxic substances [91]. The relative abundance of these two categories among the NCPs suggests an important role for detoxification and/or production of toxic SSMs in host-species adaptation. The additional presence of SSM-associated proteins and cytochrome P450s as highly represented NCPs reinforces this conclusion. In addition to MFS, several other categories of NCP transporters are known to be involved in sensing of nutritional and other environmental factors. For example, the largest single category of NCP transporters was the Ankyrin-B class, which functions to link the cytoskeleton to a variety of membrane proteins, some of which may act as receptors for plant signals [92]. The prominence of these classes among the NCP receptors suggests a necessity for adaptive changes in the sensory receptors of the pathogens to variations in the signals provided by each host plant.

Transcription factors (TFs) were another conspicuous category among the NCPs. Both species encoded non-conserved (NC) TFs belonging to two Pfam categories: PF00172 (fungal Zn(2)-Cys(6) binuclear cluster domain); and PF04082 (fungal specific transcription factor domain). A little over one third of the NC TFs were predicted to localize to mitochondria, and most of the rest to the nuclei. In *C. graminicola*, one of the predicted nuclear NC TFs was related to DEP6, which is part of the depudecin PKS gene cluster in *Alternaria brassicicola*. When DEP6 was knocked out it resulted in a small reduction in virulence on cabbage [93]. This TF gene in *C. graminicola* is part of a PKS SSM gene cluster (Cluster 28) that produces an unknown product. NC TFs in *C. sublineola* included two additional types, a bZIP transcription factor (PF00170), and two nuclear PF11951 proteins. Nearly all of these also had hits in the PHI database. One of the PF00172 proteins in *C. sublineola* was related to the CTB8 regulator of cercosporin biosynthesis in *Cercospora nicotianae*, which is part of the cercosporin gene cluster. A knock out of that gene resulted in an inability to produce cercosporin and a reduction in virulence [94]. There is a second ortholog of CTB8 in *C. sublineola* that is shared with *C. graminicola*. In *C. graminicola*, that gene is part of a PKS cluster (cluster 18) [69, 78]. However, *C. sublineola* doesn’t appear to share cluster 18, and the *C. sublineola*-specific ortholog of CTB8 was a part of a PKS cluster (cluster 11), which is not conserved in *C. graminicola* (Additional file 5: Table S6).

A third prominent category of NCPs were CAZYmes (Additional file 5: Tables S4, S5). Specific enzyme categories that were over-represented included pectinases, ligninases, and lignocellulases. Wall structures of maize and sorghum do not appear to differ very much [95, 96], so it is possible that some of these enzymes are targeted by plant defense mechanisms, which has driven their diversification [97]. Similar categories of CAZYmes were also evolving rapidly among a larger group of more distantly related genera of *Colletotrichum* fungi [25, 64].

### *Colletotrichum graminicola* and *C. sublineola* each encode non-conserved SSM-associated genes and gene clusters that may produce novel metabolites

#### Identification of SSM-associated genes in *C. sublineola* strain CgSl1

The program Ortho-MCL and the refiner COCO-CL were used to identify genes in *C. sublineola* that were orthologous to the previously identified SSM-associated genes of *C. graminicola* and *C. higginsianum* [69]. Using this approach, combined with manual annotation, 31 PKS genes, eight NRPS genes, six PKS-NRPS hybrid genes, 14 TS genes, and eight DMAT genes, were identified in *C. sublineola* (Table 7). Pfam analysis of the *C. sublineola* protein predictions identified 172 putative SSM domains. All of the SSM-associated genes that were identified by Ortho-MCL and COCO-CL (above) were included among the SSM genes identified after manual annotation of the Pfam domains. However, the Pfam analysis identified additional genes in some classes (three TSs, and one DMAT) encoded by *C. sublineola* that were not found in either *C. graminicola* or *C. higginsianum* (Table 7).

**Table 7:** Ortho-MCL prediction of shared secondary metabolism-associated genes for the three species of *Colletotrichum*

Type	*C. sublineola*	*C. graminicola* ^a^	*C. higginsianum* ^a^
PKS	31 (31)	36	36
NRPS	8 (8)	7	12
PKS-NRPS	6 (6)	4	3
DMAT	8 (9)	7	10
TS	14 (17)	14	17

*PKS* polyketide synthases, *NRPSs* non-ribosomal peptide synthetases, *PKS-NRPS hybrids* contain at least one PKS and one NRPS domain, *DMAT* dimethylallyl transferases, and *TS* terpene synthases. The numbers in parentheses represent the total number of genes in each category based on Pfam predictions for *C. sublineola* strain CgSl1. ^a^ Manual annotation was performed on data retrieved from [69]

#### Phylogenetic analysis of the SSM-associated proteins

A phylogenetic analysis was performed to address the relationships among the putative SSM-associated proteins in *C. graminicola* and *C. sublineola*. The more distantly-related species *C. higginsianum* was also included for comparison. SSM-associated genes in *C. graminicola* and *C. higginsianum* were previously published [69]. After manual annotation and identification of overlapping gene models, the 58 PKS genes that were previously identified in *C. higginsianum* [69] were reduced to 36 complete genes for analysis (Table 7). The adenylation domain (A domain) of NRPS proteins and PKS-NRPS hybrids [98, 99], the keto-synthase (KS) N-terminal and C-terminal domains of PKS proteins and PKS-NRPS hybrids [100], and the entire DMAT and TS protein sequences, were used for the phylogenetic analyses.

**Fig. 6:**
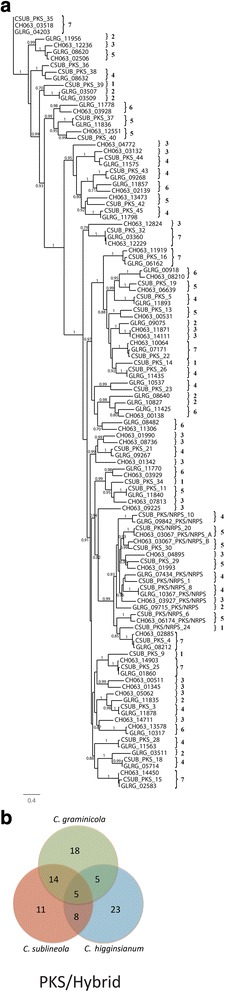
**a** Phylogenetic tree of the ketoacyl CoA synthetase domain amino acid sequences of putative PKSs and PKS-NRPS hybrids. Sequences were aligned by using MUSCLE version 3.7, and phylogenies were inferred by maximum-likelihood using PhyML version 3-0 Statistical. The numbers on the branch nodes indicate support values above 50%, calculated by aLRT. Sequences present in (1) *C. sublineola* only; (2) *C. graminicola* only; (3) *C. higginsianum* only; (4) *C. sublineola* and *C. graminicola*; (5) *C. sublineola* and *C. higginsianum*; (6) *C. graminicola* and *C. higginsianum*; and (7) *C. sublineola*, *C. graminicola* and *C. higginsianum* are indicated by the numbered brackets on the figure. **b** Venn diagram summarizing the numbers of conserved and non-conserved sequences among the three species

**Fig. 7:**
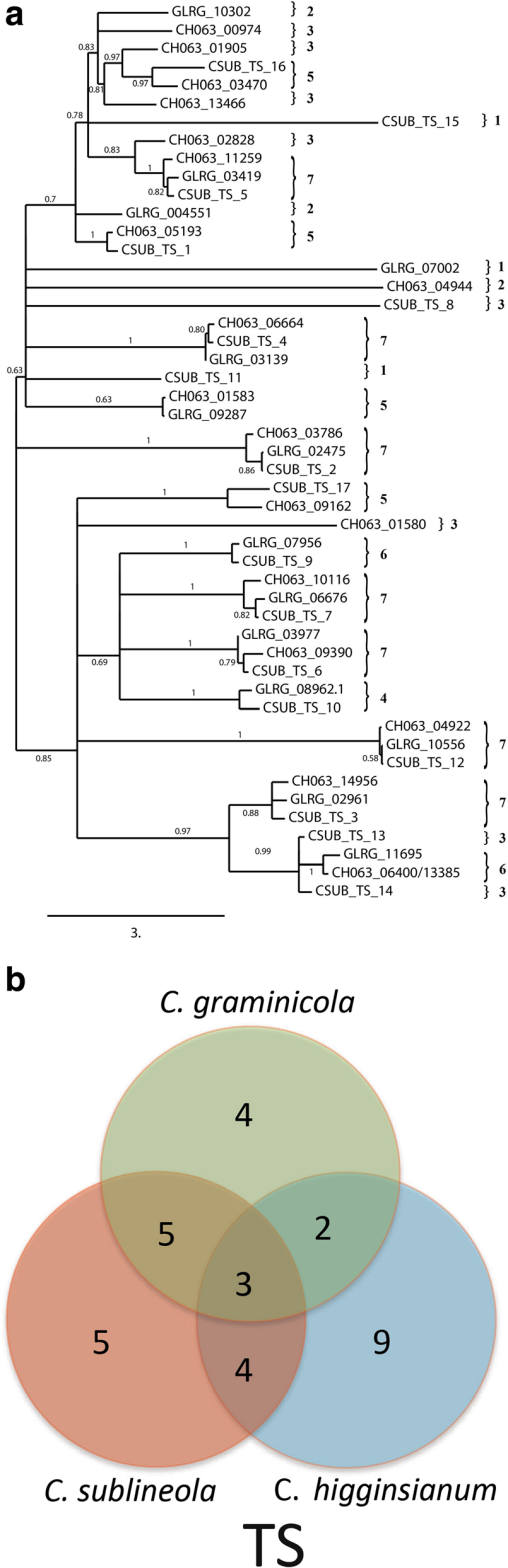
**a** Phylogenetic tree of the terpene synthase amino acid sequences. Sequences were aligned by using MUSCLE version 3.7, and phylogenies were inferred by maximum-likelihood using PhyML version 3-0 Statistical. The numbers on the branch nodes indicate support values above 50%, calculated by aLRT. Sequences present in (1) *C. sublineola* only; (2) *C. graminicola* only; (3) *C. higginsianum* only; (4) *C. sublineola* and *C. graminicola*; (5) *C. sublineola* and *C. higginsianum*; (6) *C. graminicola* and *C. higginsianum*; and (7) *C. sublineola*, *C. graminicola* and *C. higginsianum* are indicated by the numbered brackets on the figure. **b** Venn diagram summarizing the numbers of conserved and non-conserved sequences among the three species

**Fig. 8:**
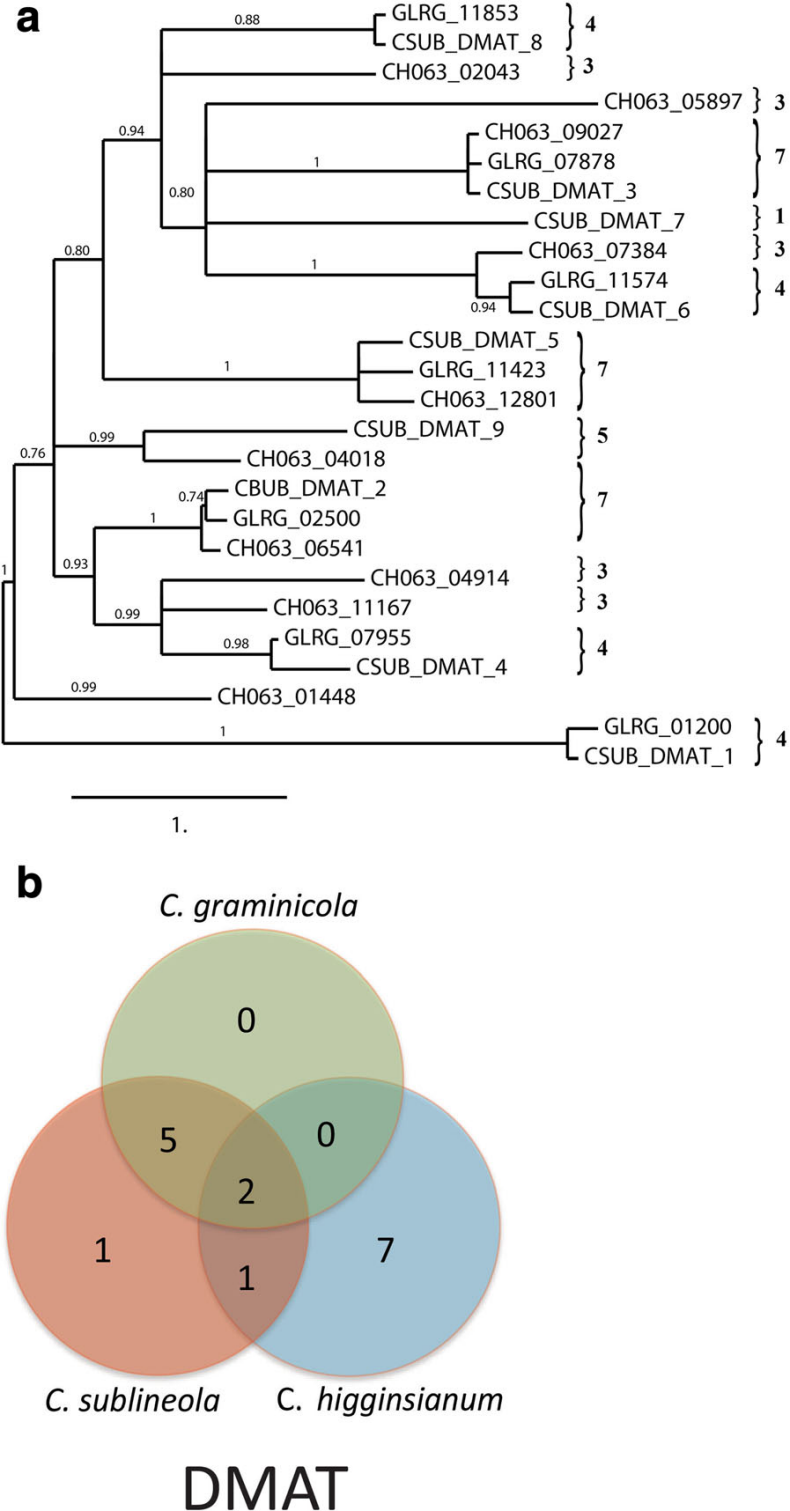
**a** Phylogenetic tree of the dimethylallyl transferase amino acid sequences. Sequences were aligned by using MUSCLE version 3.7, and phylogenies were inferred by maximum-likelihood using PhyML version 3-0 Statistical. The numbers on the branch nodes indicate support values above 50%, calculated by aLRT. Sequences present in (1) *C. sublineola* only; (2) *C. graminicola* only; (3) *C. higginsianum* only; (4) *C. sublineola* and *C. graminicola*; (5) *C. sublineola* and *C. higginsianum*; (6) *C. graminicola* and *C. higginsianum*; and (7) *C. sublineola*, *C. graminicola* and *C. higginsianum* are indicated by the numbered brackets on the figure. **b** Venn diagram summarizing the numbers of conserved and non-conserved sequences among the three species

**Fig. 9:**
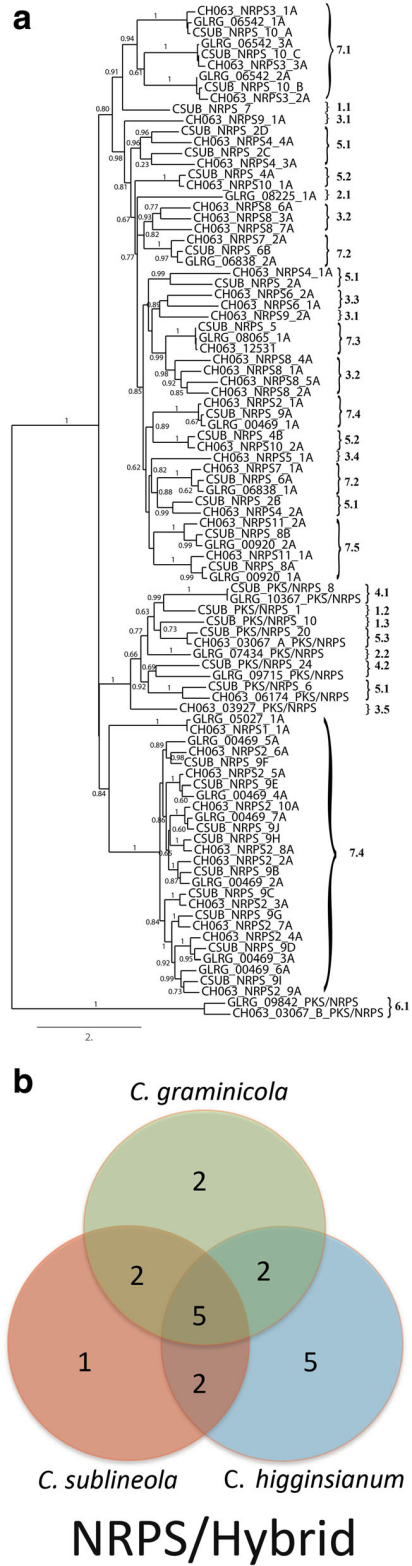
**a** Phylogenetic tree of the AMP binding domain amino acid sequences of putative NRPS and PKS-NRPS hybrids. Sequences were aligned by using MUSCLE version 3.7, and phylogenies were inferred by maximum-likelihood using PhyML version 3-0 Statistical. The numbers on the branch nodes indicate support values above 50%, calculated by aLRT. Sequences present in (1) *C. sublineola* only; (2) *C. graminicola* only; (3) *C. higginsianum* only; (4) *C. sublineola* and *C. graminicola*; (5) *C. sublineola* and *C. higginsianum*; (6) *C. graminicola* and *C. higginsianum*; and (7) *C. sublineola*, *C. graminicola* and *C. higginsianum* are indicated by the numbered brackets on the figure. The number after the period indicates modules of the same gene. **b** Venn diagram summarizing the numbers of conserved and non-conserved sequences among the three species

Results of the analysis revealed a high degree of diversity, with relatively few SSM-associated protein ortholog families that were conserved across all three *Colletotrichum* species (Figs. 6, 7, 8 and 9). As expected, *C. graminicola* and *C. sublineola* shared more ortholog families than either shared with *C. higginsianum*, consistent with a more recent common ancestor. The presence of some ortholog families only in *C. higginsianum* and *C. graminicola*, or only in *C. higginsianum* and *C. sublineola*, suggested that some members of these families may have been lost since the divergence of *C. higginsianum* from the other two species. The PKS proteins were the largest and most diverse group of SSM-associated proteins, with 79 proteins or protein ortholog families across the three species. The NRPS proteins comprised the smallest group, with only 15 different proteins or ortholog families. *Colletotrichum graminicola* and *C. sublineola* shared about half of their PKS proteins, and also about half of their PKS-NRPS hybrid and TS proteins. The DMAT and NRPS proteins were more highly conserved, with about two thirds represented in both species. Searches of the NCBI nr database, and of the predicted proteomes of five close relatives in the JGI database, revealed that there were no SSM-associated protein genes in either *C. sublineola* or in *C. graminicola* that were unique to either species (Additional file 5: Tables S4, S5).

#### Conservation of gene clusters

**Fig. 10:**
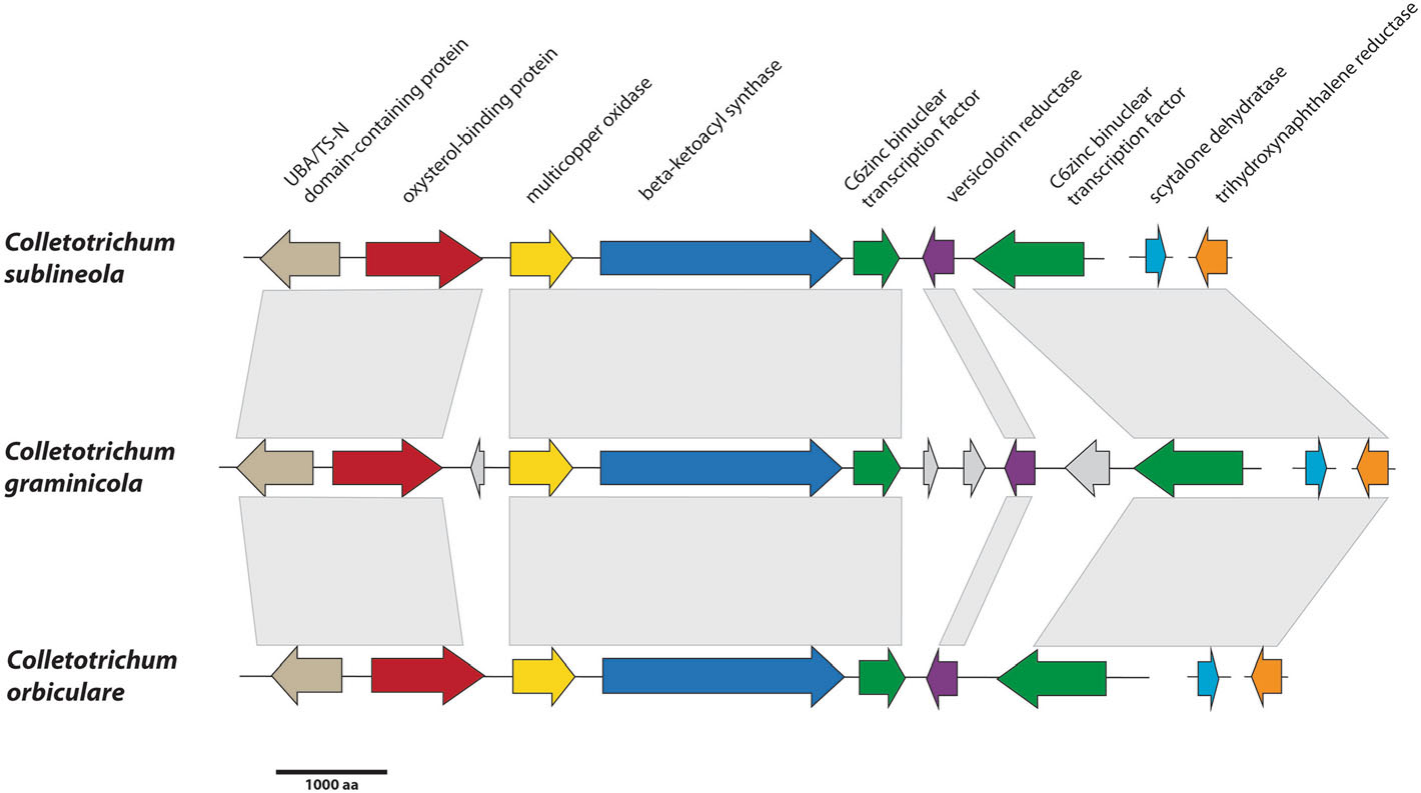
The organization of the conserved melanin gene clusters from *C. sublineola, C. graminicola* and *C. orbiculare* are shown, with orthologous genes depicted in the same color. The predicted genes shown in *gray* encode hypothetical proteins. Microsynteny among the clusters is indicated by the *gray* bars

**Fig. 11:**
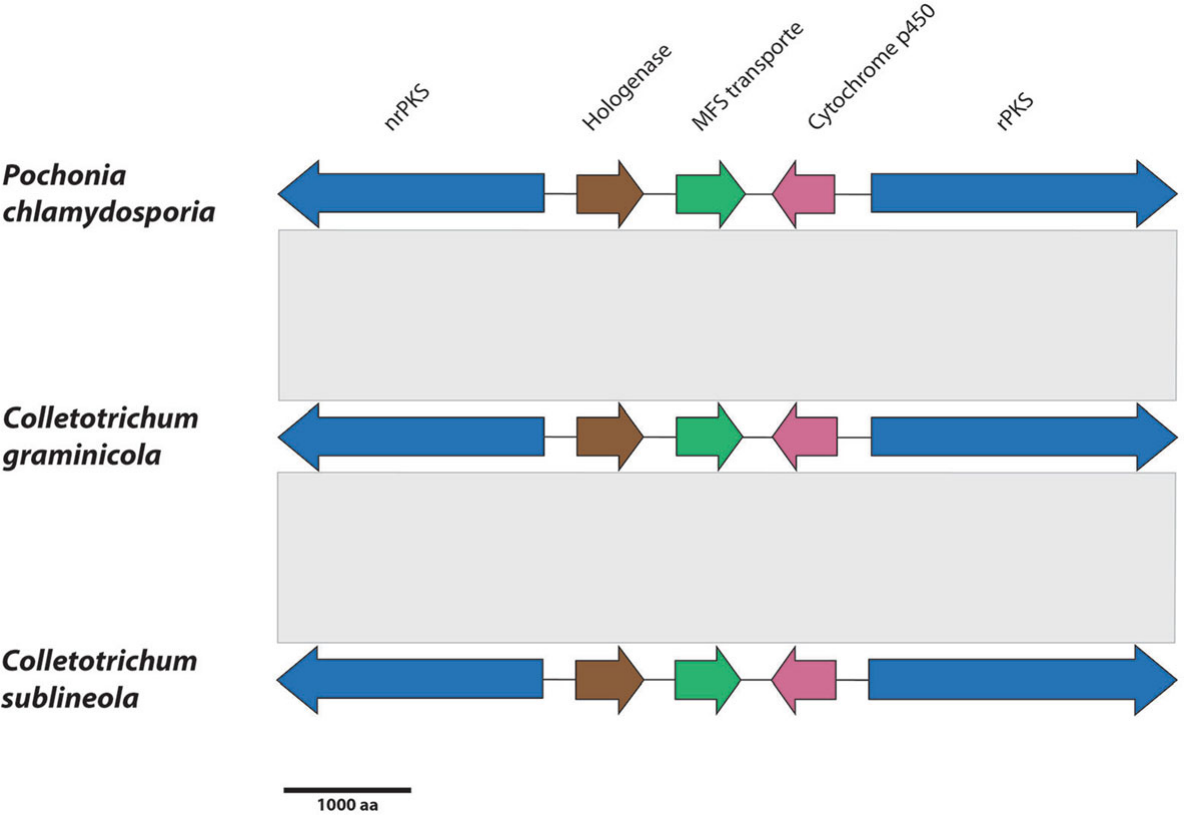
The organization of radicicol (RADS) gene clusters from *Pochonia chlamydospora, C. graminicola and C. sublineola* are shown, with orthologous genes depicted in the same color. Microsynteny among the clusters is indicated by the gray bars

Gene clusters in *C. sublineola* were identified by manual analysis of the genes located on either side of the “backbone” SSM-associated genes (ie. the genes encoding PKS, NRPS, TS, DMAT, and PKS-NRPS hybrids) that had been identified by using Ortho-MCL/COCO-CL and Pfam. A total of 67 putative SSM-associated gene clusters in the *C. sublineola* genome (Additional file 5: Table S6), were compared with the 42 clusters that were previously identified from *C. graminicola* [69]. There were 25 PKS gene clusters that appeared to be shared (with more than 50% of the genes in common) between *C. sublineola* and *C. graminicola*. One of these is the melanin cluster (Fig. 10) [69], and another is likely to be responsible for the production of monorden because it is identical in gene structure and content with the RADS cluster of *Pochonia chlamydospora* (Fig. 11) [78]. *Colletotrichum sublineola* and *C. graminicola* also shared five DMAT clusters, five NRPS gene clusters, and thirteen TS gene clusters (Additional file 5: Table S6)*.* One of these conserved TS clusters is probably involved in the production of carotenoids [69].

### *Colletotrichum graminicola* and *C. sublineola* each encode unique putative secreted proteins and SSPs

#### Identification of SSP genes in *C. sublineola* and *C. graminicola*

**Fig. 12:**
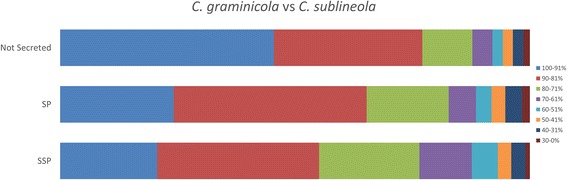
Percent similiarity among proteins that are predicted to be secreted, versus among all predicted proteins, in *C. graminicola* and *C. sublineola*

The primary characteristic for bioinformatic identification of an effector protein is that it includes an N-terminal sequence that targets it for processing and secretion. About 14% of the predicted proteins in *C. graminicola* and in *C. sublineola* had canonical signal peptides. Secreted effector proteins are usually described as small, but various sources have defined “small” differently, ranging from < 400 amino acids [101] to < 100 amino acids [102]. We chose a cutoff of 300 amino acids for our definition of SSPs. *Colletotrichum graminicola* is predicted to encode 687 small secreted proteins (SSPs) of 40 to 300 amino acids in size, with or without predicted functional domains. The number for *C. sublineola* is 824*.* The level of amino acid similarity of homologous secreted proteins is less than that of non-secreted proteins (Fig. 12). If only SSPs are considered, versus all secreted proteins, the level of similarity is even lower (Fig. 12).

**Fig. 13:**
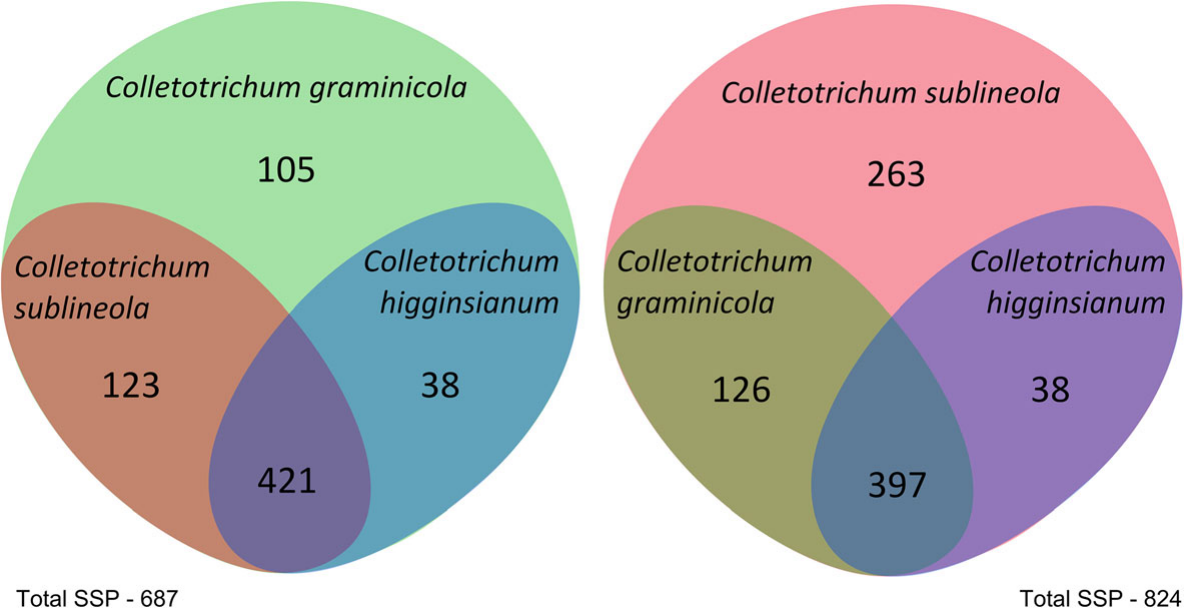
Venn diagrams summarizing conservation of predicted small-secreted proteins among *C. graminicola*, *C. sublineola*, and *C. higginsianum*. Shared proteins were identified by the Reciprocal Best Hit (RBH) method

**Fig. 14:**
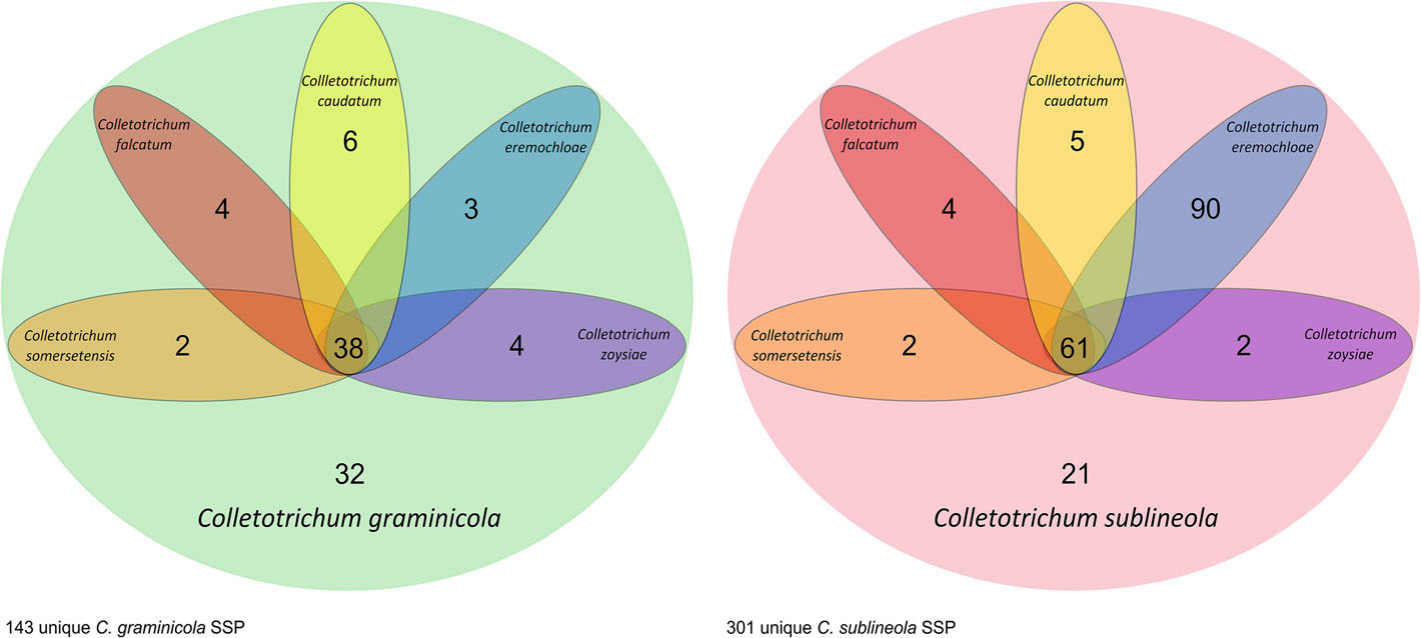
Venn diagrams summarizing conservation of predicted small-secreted proteins of *C. graminicola* strain M1.001 and *C. sublineola* strain CgSl1 with five close relatives. Shared proteins were identified through a combination of blastp and tblastn searches of the predicted proteomes and translated assemblies (respectively) of these five relatives with the protein sequences from *C. graminicola* and *C. sublineola*

*Colletotrichum graminicola* and *C. sublineola* have more SSPs in common than either share with their more distant relative *C. higginsianum* (Fig. 13). *Colletotrichum graminicola* M1.001 encodes 143 predicted SSPs that are not found in *C. sublineola* strain CgSl1, while *C. sublineola* has 301 that are not shared with *C. graminicola* (Additional file 5: Tables S4, S5). The majority of these NC SSPs from both species (67% in *C. graminicola*, and 66% in *C. sublineola*) were similar to predicted proteins in other fungi in the NCBI database, although in most cases these were classified as hypothetical proteins (Additional file 5: Tables S4, S5). The remainder in each case did not match predicted protein sequences from any other species in the NCBI nr database. Analysis with the EffectorP prediction tool [103] revealed that about 60% of the NC SSPs in each species had a probability of at least 50% of being fungal effectors (Additional file 5: Tables S4 and S5). After additional comparisons with the available genome data from a group of five close relatives of *C. graminicola* and *C. sublineola* (http://genome.jgi.doe.gov/), there appeared to be only 32 *C. graminicola* LS-SSPs, and 21 *C. sublineola* LS-SSPs (Fig. 14). Interestingly, *C. sublineola* shares more SSPs with *C. eremochloae* than it does with any of the other close relatives included in the JGI database. *Colletotrichum eremochloae* is a pathogen of centipedegrass, and it was previously shown to be very closely related to *C. sublineola* [104].

**Fig. 15:**
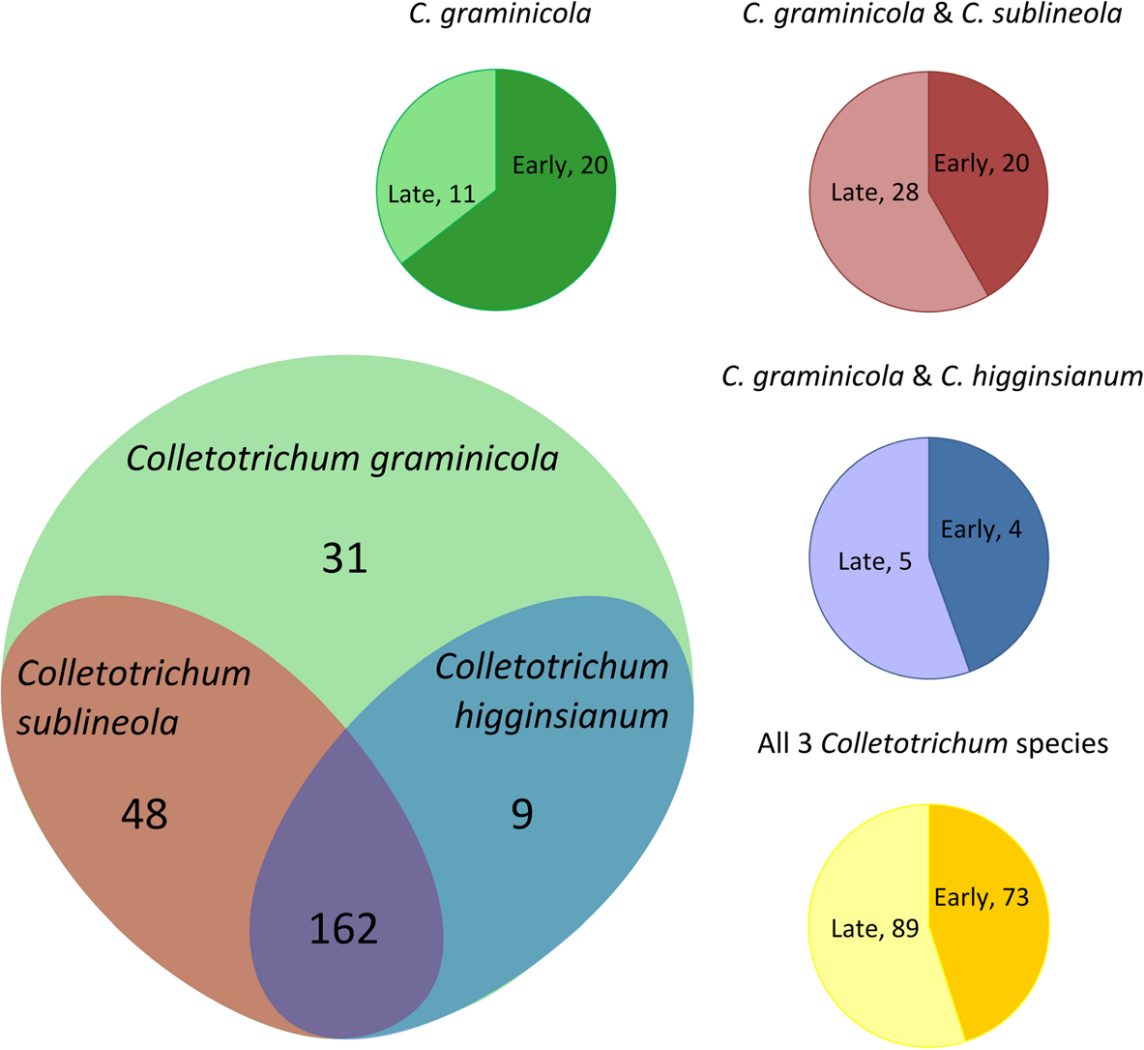
Expression patterns of SSP-encoding genes that matched transcripts from the *in planta C. graminicola* transcriptome [78]

Analysis of *C. graminicola in planta* transcriptome data [78] revealed that a majority of the transcribed *C. graminicola* NC SSP genes were more highly expressed in the early stages of infection (appressoria and/or biotrophy), whereas less than half of the genes shared with *C. sublineola* and/or with *C. higginsianum* were expressed during these early stages (Additional file 5: Table S4, Fig. 15).

#### Characterized effector classes among NC SSPs

Several classes of fungal effectors described in the literature from other organisms are included among the NC SSPs of *C. graminicola* and *C. sublineola*.

The CFEM proteins have an eight cysteine-containing domain of around 66 amino acids [88]. Some CFEM proteins have important roles in pathogenesis [105, 106]. There are 11 CFEM SSPs in *C. graminicola* M1.001, and *C. sublineola* CgSl1 has homologs for 10 of these (Additional file 5: Tables S1 and S2). The *C. sublineola* epitype strain S3.001 has a homolog for the eleventh (http://genome.jgi.doe.gov/).

Effectors with chitin-binding domains [107] are thought to bind to chitin present in fungal cell walls, thus protecting the pathogen from plant chitinases [108]. *Colletotrichum graminicola* and *C. sublineola* share two SSP genes that encode chitin binding domains (Additional file 5: Table S1). *Colletotrichum graminicola* encodes one additional NC chitin-binding SSP (Additional file 5: Table S4).

Genes containing lysin motifs (LysM) are conserved in pathogenic and nonpathogenic fungi [109]. They appear to be highly divergent among species, and thus to be evolving rapidly [102]. LysM effectors, eg. Ecp6 from *Cladosporium fulvum*, are believed to sequester fungal chitin fragments, thus preventing host detection [110–112]. In *C. lindemuthianum*, a LysM protein called ClH1 was localized specifically to the surface of biotrophic hyphae by using a monoclonal antibody [113, 114]. There are two predicted LysM-domain SSP genes in *C. graminicola,* one of which is a homolog of ClH1. Both of these are expressed during the early stages of fungal colonization in the WT strain (Additional file 5: Table S4). *Colletotrichum sublineola* has four predicted LysM-domain SSP genes. Two of these are shared with *C. graminicola*, including a homolog of C1H1.

There are five predicted *C. graminicola* proteins, and nine in *C. sublineola*, that belong to the conserved NEP1-like protein (NLP) family [85], which also includes the NPP1 family of *Phytophthora* effectors [115]. This family induces apoptosis in host plant tissues, and members are believed to play roles in the induction of necrotrophy [116–118]. Four NLPs are conserved in the two *Colletotrichum* species, and also have homologs in *C. higginsianum* [102]. In *C. higginsianum*, two of five NLPs (ChNLP3 and ChNLP5) lacked crucial amino acids and were not able to induce necrosis in *N. benthamiana* [102]. There are two putative *C. sublineola* homologs of ChNLP3 and three of ChNLP5, but *C. graminicola* has only a single homolog for each of these proteins. Two additional SSPs containing NPP1 domains in *C. sublineola* are not conserved in *C. graminicola* (Additional file 5: Table S5).

Only 21 *C. graminicola* NC SSPs, and 46 *C. sublineola* NC SSPs, matched Pfam categories. The vast majority (117 in *C. graminicola* and 225 in *C. sublineola*) did not have Pfam classifications, and this group included all of the LS-SSP proteins.

#### SSP families

The existence of gene families was explored by using blastp to identify potential orthologs and paralogs among the SSPs from *C. graminicola* and *C. sublineola*. The 1511 SSPs from the two species could be grouped into 789 families of related sequences (Additional file 5: Table S7). Most of the 325 conserved families that included members from both species were comprised of only one member in *C. graminicola* and one in *C. sublineola*. About 1/3 of the conserved families consisted of more than one putative paralog in one or both species. The largest conserved family included 29 predicted glycosyl hydrolase genes; 14 paralogs in *C. graminicola*, and 15 in *C. sublineola*.

*C. graminicola* had 189 NC SSP gene families that were not found in *C. sublineola*, and *C. sublineola* had 275 that were not found in *C. graminicola* (Additional file 5: Table S7). Among these NC families, nine included two paralogs, while the rest were each represented by only a single member. None of the NC families included more than two members. These results suggest that there has been relatively little duplication of SSP proteins within these two species.

#### SSP and SSM diversity among isolates

We sequenced the genome of a second strain of *C. graminicola*, M5.001, which was isolated from maize with anthracnose symptoms in the late 1980s in Brazil. This strain is sexually compatible with M1.001 [119]. Assembly and annotation statistics are included in Table 1, and predicted protein sequences are provided in Additional file 6. Only 73 out of the 12006 M1.001 predicted gene sequences (~1%) had no match in the M5.001 assembly (Additional file 5: Table S4). Only five of those genes were predicted to encode SSPs, while one was a putative SSM-associated gene. Of the 73 predicted M1.001 strain-specific genes, only seven had no matches to any other sequences in the NCBI nr database or the JGI databases (Additional file 5: Table S4). None of these seven had Pfam descriptions, and none were predicted to encode SSPs or SSM-associated proteins. There was transcript evidence for only one of them (Additional file 5: Table S4). The apparent low number of strain-specific SSPs in *C. graminicola* is consistent with an earlier report [120] that suggested that differences in expression may be more important than presence-absence polymorphisms for pathotype identity.

Two other genome assemblies are available for *C. sublineola*. The TX430BB strain was isolated in Texas in the late 1980s, and was sequenced by Baroncelli et al. [25]. The S3.001 strain is the epitype for the species [10, 104], and its genome assembly can be accessed from JGI (http://genome.jgi.doe.gov/). This strain was isolated in the late 1980s in Burkina Faso [5].

*C. sublineola* isolate CgSl1 has 117 predicted gene sequences (<1%), including 23 SSP genes, that are not found in the TX430BB assembly (Additional file 5: Table S5). It has 147 gene sequences (~1%) that are not found in S3.001, only 7 of which encode SSPs. Only 39 gene sequences are not found in either of the other two other strains, including 2 SSPs. All of the SSM-associated genes in CgSl1 appear to have matches in both other strains of *C. sublineola*. Of the 39 CgSl1 strain-specific genes, only four had no matches to any other sequences in the NCBI nr database or the JGI databases (Additional file 5: Table S5). None of these genes encodes an SSP, and only one has a Pfam domain (PF12511, a protein of unknown function).

The apparent rarity of strain-specific SSP gene sequences differs from some other fungal species, eg. *Magnaporthe oryzae,* where the deletion of secreted effector genes seems to be common, and to play an important role in the evolution of new races [121, 122]. However, comparisons with genome assemblies of the five closely related species within the graminicolous clade, accessed from JGI (http://genome.jgi.doe.gov/), suggests a more important role for deletion of effector genes, as well as other classes of genes, in speciation and host species adaptation, a finding that has also been reported by others based on comparative analyses of a wider range of *Colletotrichum* genera [25, 64].

## Conclusions

In this work we have compared gene models from two contemporaneous, co-occurring strains of the sibling species *C. graminicola* and *C. sublineola*, and identified those that do not appear to be conserved as potential candidates for involvement in host specificity. Our approach was based on previous studies that have shown that gene gain and loss is associated with host range in many plant pathogens, including *Colletotrichum* [25, 64]. However, we do not mean to suggest that products of conserved genes don’t also play important roles, either alone or in combination with non-conserved gene products, in host specialization. The list of non-conserved genes identified in this work is a function of how we defined them, including the level of similarity that we considered significant, and the ability to accurately assign orthologs.

Our analysis confirmed that the genomes of the *C. graminicola* and *C. sublineola* strains were very similar to one another in both gene content and gene order, consistent with a relatively recent common ancestor. We also confirmed that each strain was able to successfully colonize its own living host (maize and sorghum, respectively), while the closely related non-host underwent an apparent hypersensitive response upon challenge. After applying our chosen parameters, we found that 14% of the *C. graminicola* gene models, and 22% of the *C. sublineola* gene models, were not conserved in the other species. Certain categories of genes were especially likely to be non-conserved including, as expected, genes that were predicted to encode SSPs and SSM-associated proteins that may play important roles in early events related to host recognition and the induction of compatibility. A relatively small number of the NC SSP gene sequences were also not conserved among different strains within each species, especially *C. sublineola*, which suggested the possibility of selection within the population and a potential Avr function. Races of both *C. sublineola* and *C. graminicola* have been reported to occur [123–130].

The majority of NCPs were not SSPs or SSM-associated proteins. Transporters, cytochrome P450s, and signaling proteins were well-represented, suggesting an important role for these functions in adaptation to varying aspects of each host environment, and in the secretion or evasion of toxic secondary metabolites. Transcription factors were also particularly abundant, suggesting that changes in gene expression patterns may be more important than the presence/absence of individual genes. Transcriptome and proteome comparisons would help us to address this hypothesis. CAZYmes were another common category, in spite of similarity of cell wall structure in maize and sorghum. It is known that some plant defenses target some CAZYmes in the apoplast [97] so it may be that these CAZYmes have diversified as a result of selection against host specific defenses. A relatively large number of the NCPs in both species were not categorized by either Ortho-MCL or Pfam. Many of these genes appeared to be conserved in other fungi, where they are predicted to encode hypothetical proteins of unknown function. Many are predicted to be secreted, or targeted to the nucleus or the mitochondria, and may interact with specific host factors to suppress or avoid host defenses, or to establish biotrophic hyphae or nutritional access. Similar categories of proteins were found to be rapidly evolving among several more distantly related *Colletotrichum* genera, suggesting that these categories play important roles in niche adaptation across the entire genus [64].

Our findings indicate that host specificity in these closely related pathosystems is not only a matter of recognition of, and response to, particular pathogenicity factors at the point of attempted penetration. Differences in fungal gene content reflect a much broader adaptation to the living host environment across the entire course of pathogen development, which has presumably developed during co-evolution of the host and its pathogen.

We found that the quality of the available assemblies and annotations had an important impact on our findings. We compared the published Broad annotation of *C. graminicola* with our MAKER annotation of *C. sublineola*. According to these data, *C. sublineola* had more genes than *C. graminicola*. As an exercise, we re-annotated *C. graminicola* with MAKER, and 14,419 genes were predicted, 1,108 more than MAKER predicted for *C. sublineola*. Comparison of the two annotations of *C. graminicola* (MAKER and Broad) using blastp revealed that they had about 10,000 genes in common, while the rest of the gene models were specific to each annotation (Additional file 5: Table S8). Some of the genes that were found in only one annotation were predicted to encode SSPs or SSM-associated proteins (Additional file 5: Table S8). We conclude from this exercise that the total number of potential SSP and SSM-associated genes we have reported here for *C. graminicola* and *C. sublineola* might be under-estimated, while the numbers of unique SSPs and SSM-associated proteins could be somewhat inflated. When we mapped the potential unique genes from each species against the genome assemblies of the other, between 50 and 70% of these genes did not hit the assembly of the other strain at all, and thus do appear to be truly non-conserved sequences. Among the apparently NC genes that did have hits to the assembly, our preliminary investigations suggest that many were not annotated due to fragmentation, which may be related to the different assembly qualities. The *C. graminicola* assembly, which was produced by using a combination of Sanger and 454 sequencing, includes fewer contigs and scaffolds than the *C. sublineola* assembly, which was done by using 454 alone. This fragmentation effect is expected to become progressively more significant as methods providing shorter reads (eg Illumina) are increasingly used for genome sequencing in fungi. Although it has not been widely acknowledged in previous comparative studies, it is clear that the use of datasets from diverse sources that have been developed by using different assembly and annotation programs and program parameters will have an impact on the results. Because of this, we emphasize the importance of confirming these data with other methods (e.g. amplification and cloning of entire genes, and confirmation of absence by hybridization or sequencing analysis), before proceeding with any additional studies focused on individual genes.

This work has provided important clues to functions (i.e. detoxification and transport, regulation of host and pathogen gene expression, and signaling and recognition) that are important in the determination of host preference among these two closely related and economically important pathogens. The data included here will provide a useful foundation for further studies to explore the basis for non-host recognition, with the goal of using this information to develop improved varieties of maize and sorghum for management of anthracnose diseases.

## Methods

### Plant and fungal growth and inoculation

Strains M1.001 and CgSl1 were originally obtained from Drs. Ralph Nicholson and Bob Hanau (Purdue University) and preserved on silica gel at −80 °C [131]. They are available from the corresponding author by request. Strains were cultured on potato dextrose agar (PDA, BD Difco, Franklin Lakes, NJ) under continuous fluorescent light at 23 °C. Spores were harvested from 2-week-old culture plates by gently scraping them from the surface, and washed three times before use.

Sweet sorghum variety Sugar Drip was obtained from Dr. Todd Pfeiffer (University of Kentucky). Maize inbred Mo17 was obtained from the North Central Regional Plant Introduction Station. Seeds were sown in a mixture of two parts sterile topsoil and three parts of Pro-Mix BX (Premiere Horticulture, Ltd, Riviere du Loup, PQ, Canada). Seedlings were maintained in the greenhouse with 14 h of light, watered every other day to saturation using an automated overhead irrigation system, and fertilized beginning 1 week after emergence two or three times per month as needed with a solution of 150 ppm of Peters 20-10-20 (Scotts-Sierra Horticultural Product Co., Marysville, OH).

Maize leaf sheaths were inoculated with a suspension of 5 × 10^5^ spores per ml as described in [16]. Sorghum leaf sheaths were inoculated with a similar protocol, but instead of applying a single drop of inoculum, the leaf sheaths were entirely filled with the spore suspensions. Maize and sorghum seedlings at the V6 stage were inoculated with a suspension of 5 × 10^6^ spores per ml by using a compressed-air spray applicator (Preval Model 267 Paint Spray Gun). After inoculation, the plants were incubated for 18 h in the dark at 25 °C in a dew chamber at 100% relative humidity before being returned to the greenhouse bench.

### Sequencing and assembly of fungal genomes

Genomic DNA was extracted from fungal cultures by using the method described in [69] Shotgun Libraries were prepared according to the “Rapid Library Preparation Method Manual” (2010) for the GS FLX Titanium Series, using the Library Prep Kit with Rapid Library Rgt/Adaptors (Roche, Pleasanton CA). Paired-End 3000 Libraries were prepared according to the “GS FLX Titanium 3 kb Span Paired End Library Preparation Method Manual” using a Library Prep Kit with General Library Reagents and the GS FLX Titanium Paired End Adaptor Set (Roche). Emulsion PCR and enrichment was performed according to the “GS FLX emPCR Method Manual“ using the emPCR Kit Reagents (Lib-L) (Roche). Beads were loaded onto a PicoTiterPlate (70 × 75) for sequencing with the Sequencing Kit Reagents XLR70 (Roche). The genomes of *C. graminicola* strain M5.001 and *C. sublineola* strain CgSl1 were sequenced to 29X, and 43X coverage, respectively. Genome assembly was done by using Newbler version 2.9. The M5.001 Whole Genome Shotgun project has been deposited at DDBJ/ENA/GenBank as BioProject SAMN06043298, under the accession number MRBI00000000. The version described in this paper is MRBI01000001. The CgSl1 Whole Genome Shotgun project has been deposited at DDBJ/ENA/GenBank as BioProject PRJNA356071, under the accession number MQVQ00000000. The version described in this paper is MQVQ01000001.

The genome assemblies for *C. graminicola* strain M1.001 and for *C. sublineola* strain TX430BB were downloaded from the NCBI BioProjects database (BioProjects PRJNA37879 and PRJNA246670, respectively). Genome assemblies for *C. sublineola* strain S3.001 and for *C. falcatum, C. somersetensis, C. caudatum, C. eremochloae,* and *C. zoysiae* were downloaded from the Joint Genomes Institute Genome Portal (http://genome.jgi.doe.gov/).

### Comparative analysis of genome assemblies

The genome assemblies were repeat-masked using a filtering algorithm previously implemented in TruMatch [132] The masked genomes were then aligned with one another in reciprocal pairwise using blastn with an e-value cutoff of 1e-200. The resulting blast reports were pre-screened to filter out aligned regions that contain hidden paralogs and single nucleotide polymorphisms were then identified. Finally, the SNP totals were divided by the total length of uniquely aligned sequence and multiplied by one million to provide a standard measure of genetic distance (SNPs/Mb). All steps in the analysis are implemented in a package of perl scripts known as SNPcounts.pl (available on request).

### Genome annotation

The *C. sublineola* CgSl1 genome was annotated by using MAKER version 2.03 (http://www.yandell-lab.org/software/maker.html). Assembled contigs were filtered against RepBase model organism “fungi” with RepeatMasker version open-3.2.8. The MAKER analysis used the ab initio gene predictors AUGUSTUS version 2.3.1 (*Fusarium* model), GeneMark-ES version bp 2.3a (self-trained, see below), and SNAP version 2006-07-28 (self-trained, see below). Supporting evidence provided to MAKER consisted of protein sequences from *Colletotrichum graminicola* M1.001, as previously published [69]; and normalized unigenes from *C. graminicola* M1.001 as alternate organism EST evidence. To allow identification of previously-unannotated genes, MAKER was instructed to retain ab initio predictions that were not concordant with this evidence. MAKER was also instructed to extend coding sequences to include start and stop codons.

The *C. graminicola* M5.001 and M1.001 genomes were annotated by using MAKER version 2.28 (http://www.yandell-lab.org/software/maker.html). Assembled contigs were filtered against RepBase model organism “fungi” with RepeatMasker version open-3.2.8. The MAKER analysis used the ab initio gene predictors AUGUSTUS version 2.3.1 (*Fusarium* model), FGENESH version 3.1.1 (*Fusarium* model), GeneMark-ES version bp 3.9e (self-trained, see below), and SNAP version 2006-07-28 (self-trained, see below). Supporting evidence provided to MAKER included all complete protein sequences from *Colletotrichum* in the NCBI non-redundant protein database. As with *C. sublineola* annotations, MAKER was instructed to retain ab initio predictions. MAKER was also instructed to take additional steps to find alternatively spliced transcripts, and to extend coding sequences to include start and stop codons.

The two self-trained ab initio predictors were trained on the gene annotations produced by a preliminary MAKER run which did not include these two predictors (that is, using only AUGUSTUS, protein evidence, and alternate organism EST evidence for *C. sublineola*; and AUGUSTUS, FGENESH, and protein evidence for *C. graminicola*). To produce annotations more suitable for training SNAP and GeneMark-ES, this preliminary MAKER run was instructed to disregard ab initio predictions not concordant with protein evidence, to disregard single-exon evidence, and not to take additional steps to find alternatively-spliced transcripts. Other than these exceptions, the preliminary training run used the same inputs and parameters as the final MAKER run.

The predicted protein sequences for *C. sublineola* strain CgSl1 that were used for this work are included as supplementary data (Additional file 3). The predicted protein sequences for *C. graminicola* strain M5.001 are included in (Additional file 6).

### Comparative analyses of genome annotations

To identify M1.001 gene sequences that were not present in the *C. sublineolum* assembly, (Additional file 5: Table S4), nucleotide sequences from Broad gene annotations of M1.001 published previously [69] were aligned against the *C. sublineolum* genome using exonerate version 2.2.0 (model est2genome) [133] (Additional file 5: Table S4). A gene sequence was considered non-unique if there was an alignment with at least 40% of the possible score for a sequence of that length. The same procedure was used to compare *C. sublineolum* MAKER annotations to the *C. graminicola* genome assembly (Additional file 5: Table S5).

As an exercise, the MAKER annotation for M1.001 (see above) was compared with the Broad annotation published previously [69]. The set of inferred protein sequences of the MAKER annotations were aligned against the set of inferred protein sequences of the Broad annotations using NCBI BLAST version 2.2.18 in protein-to-protein (blastp) mode.

For each protein sequence *P,* the best alignment against the set of sequences annotated by the other procedure (MAKER or Broad), as determined by blastall -b 1, was selected. High-scoring pairs (HSPs) with an e-value of 1e-10 or higher were discarded, and a percent identity *ID*_*A*_ for the alignment was obtained by weighted average of the percent identities of the remaining HSPs, with the alignment length of the HSP as the weight. The total alignment length *L*_*A*_ was taken to be the sum of the alignment lengths of the (non-discarded) HSPs.

A gene was considered to be a unique annotation if the percent identity, weighted by the ratio of total alignment length to query or to target length, was less than 70%. That is, an annotation was considered unique if either *ID*_*A*_ × *L*_*A*_ /*L*_*P*_ < 70%, or *ID*_*A*_ × *L*_*A*_ /*L*_*H*_ < 70%, where *L*_*P*_ denotes the length of the query sequence *P* and *L*_*H*_ denotes the length of the sequence that was selected as the best hit among annotations produced by the other method.

### Genome synteny

Genome synteny was analyzed by using the Synteny Mapping and Analysis Program (Symap) v4.2 [134] and default parameters. *Colletotrichum sublineola* scaffolds were aligned to the 13 previously published chromosomes of *C. graminicola* strain M1.001 [69].

### Identification of orthologous and unique genes

Fungal protein sequences used in this study were downloaded from the Broad Institute (*C. graminicola, C. higginsianum, Fusarium graminearum, F. oxysporum, Verticillium dahliae, Aspergillus flavus*) and the Joint Genome Institute (*Trichoderma reesei, C. falcatum, C. somersetensis, C. caudatum, C. eremochloae, C. zoysiae, C. sublineola* strain S3.001). Protein sequences from *Epichloë festucae* were the FGENESH gene predictions previously used in the Clavicipitaceae analysis [135]. Putative orthologs were identified by using two methods. The first method was application of Ortho-MCL and COCO-CL (COrrelation COefficient-based CLustering) to the annotations [76, 136], following a procedure previously used for ortholog identification within the Clavicipitaceae [135]. The species included for comparison in the Ortho-MCL/COCO-CL analysis were: *C. graminicola*; *C. higginsianum*; *C. sublineola* CgSl1; *M. oryzae*; *E. festucae*; *F. graminearum*; *F. oxysporum*; *T. reesei*; *V. dahlia*; and *A. flavus*. The second method used for ortholog identification was Reciprocal Best Hit (RBH) with an expect-value cutoff of 1e-5 [77, 137]. This method was used to compare proteins from *C. graminicola*, *C. sublineola*, and *C. higginsianum*.

### Protein characterization

Predicted proteins were compared by blastp with the non-redundant protein sequence database from NCBI (https://blast.ncbi.nlm.nih.gov/Blast.cgi) with an expect-value cutoff of 1e-5 [138]. Predicted proteins were assigned to functional families by comparing to the Protein Family (Pfam) database (http://pfam.sanger.ac.uk/) version 29.0 (December 2015) by using pfamScan software version 1.5 (October 2013), with an e-value cutoff of 1e-5 [139]. Transporters were predicted by using the Transporters Classification Database (http://www.tcdb.org) (2016) with an e-value cutoff of 1e-5 [140]. CAZymes were characterized by using dbCAN HMMs version 5.0 (http://csbl.bmb.uga.edu/dbCAN/annotate.php), which is based on the classification scheme of CAZyDB [141, 142]. Predicted proteins were compared with the Pathogen-Host Interaction (PHI) database (www.phi-base.org) Version 4.1 (May 2016) [74, 75] using blastp and an e-value cutoff of 1e-5. To predict protein localizations, WoLF-PSORT for fungi [143] version 0.2 (August 2006) was used, as described in [69]. For the classification of putative secreted proteases, the sequences of predicted secreted proteins were submitted to MEROPS release 10.0 batch blast analysis (http://merops.sanger.ac.uk) [144] also as described [69]. For prediction of fungal effectors, predicted secreted proteins were analyzed by using the EffectorP prediction tool (http://effectorp.csiro.au) (December 2015) [103].

### SSM-associated proteins

The five classes of candidate SSM-associated genes (PKS, NRPS, PKS-NRPS hybrid, DMAT, and TS) were identified from *C. sublineola* by applying a process that included Pfam and Ortho-MCL/COCO-CL analysis; followed by manual annotation and validation of domains using the Conserved Domain Database (CDD) (http://www.ncbi.nlm.nih.gov/cdd/); blastp comparisons with the NCBI nr database; and InterproScan analysis. This protocol has been described in more detail previously [69].

*Colletotrichum sublineola* SSM gene clusters were manually annotated by evaluating Ortho-MCL/COCO-CL results for the genes that were located upstream and downstream of the SSM-associated backbone genes. Genes that had no or few orthologs were considered to belong to the clusters, while genes that were conserved in most or all of the ten species included in the analysis defined the outside boundaries of the clusters.

### Phylogenetic analysis of SSM-associated proteins

Phylogenetic analysis of SM genes was performed by using phylogeny.fr (http://www.phylogeny.fr/index.cgi) (2003). The A and KS N-terminal and C-terminal domains of the NRPS, PKS, and NRPS-PKS hybrids were identified by using the NCBI CCD. Amino acid sequences were aligned by using MUSCLE version 3.8.31 (May 2010) [145] and default parameters, and phylogenies were inferred by maximum-likelihood using PhyML version 3.0. Statistical branch support was provided by an approximation to the standard likelihood ratio test, aLRT [146].

## Additional files

Additional file 1: Figure S1. M1.001 on Sugar Drip sorghum, 48 hpi, cells beneath appressoria (white arrows) plasmolyzed (result not typical). Scale bars equal to 50 μm. (JPG 302 kb)
Additional file 2: Figure S2. Plasmolysis controls. A: Maize leaf sheath, 72 h after mock inoculation, most cells still plasmolyze. B: Sugar Drip leaf sheath, 72 h after mock inoculation, most cells still plasmolyze. Scale bars equal to 50 μm. (JPG 645 kb)
Additional file 3: FASTA predicted proteins of *C*. *sublineola* strain CgSl1. (TXT 6816 kb)
Additional file 4: Figure S3. Alignments of sequences of CgSl1 with species type S3.001. A: actin, B: chitin synthase, C: histone H3, D: beta-tubulin, E: ITS. Alignments done with MUSCLE version 3.7 and default parameters. (JPG 300 kb)
Additional file 5: EXCEL file including detailed analysis of the genes of *C. graminicola* M1.001 and *C. sublineola *CgSl1. (XLSX 1703 kb)
Additional file 6: FASTA predicted proteins of *C. graminicola* strain M5.001. (TXT 7838 kb)
