# Correction to: A comparative genomic analysis of putative pathogenicity genes in the host-specific sibling species *Colletotrichum graminicola* and *Colletotrichum sublineola*

**DOI:** 10.1186/s12864-018-5073-3

**Published:** 2018-09-19

**Authors:** E. A. S. Buiate, K. V. Xavier, N. Moore, M. F. Torres, M. L. Farman, C. L. Schardl, L. J. Vaillancourt

**Affiliations:** 1 Department of Plant Pathology, University of Kentucky, 201F Plant Science Building, 1405 Veterans Drive, Lexington, KY 40546-0312 USA; 2 Present Address: Monsanto Company Brazil, Uberlândia, Minas Gerais Brazil; 3 Department of Computer Science, University of Kentucky, Davis Marksbury Building, 328 Rose Street, Lexington, KY 40504-0633 USA; 4 Present Address: Functional Genomics Laboratory, Weill Cornell Medicine, Doha, Qatar

## Correction

Following the publication of this article [1], the authors informed us of the following error. The authors wish to make it known that they mistakenly used some unpublished data from the Joint Genome Institute (JGI) database in some parts of this article in a way that violates the JGI usage agreement. They are therefore issuing a correction to their article in which Fig. 14 and all text in the article that refers to results involving JGI data are excluded. Specifically, the sections to be disregarded are comparisons to the JGI sequences described in the section titled “Characteristics of the *C. graminicola* and *C. sublineola* NCPs”: comparisons with S3.001 from the section titled “SSP and SSM diversity among isolates”: and the summarized numbers of unique SSPs and SSMs in the abstract. The revised sentence in the abstract should read “Only 98 SSP genes appeared to be specific to *C. graminicola*, and 103 to *C. sublineola*”. It is important to note that these revisions do not change the conclusions of the paper in any meaningful way. The authors apologize to Dr. Crouch and the other principal investigators of the relevant JGI Community Sequencing Project (https://genome.jgi.doe.gov/portal/Gensignvironment/Gensignvironment.info.html) for this error.

## References

[1] Buiate EA (2017). A comparative genomic analysis of putative pathogenicity genes in the host-specific sibling species Colletotrichum graminicola and Colletotrichum sublineola. BMC Genomics.

