# Supplementary figures and images for: A comparative genomic analysis of putative pathogenicity genes in the host-specific sibling species Colletotrichum graminicola and Colletotrichum sublineola
Source: BMC Genomics. 2017 Jan 10;18:67. doi: 10.1186/s12864-016-3457-9 (PMC5225507; doi:10.1186/s12864-016-3457-9)

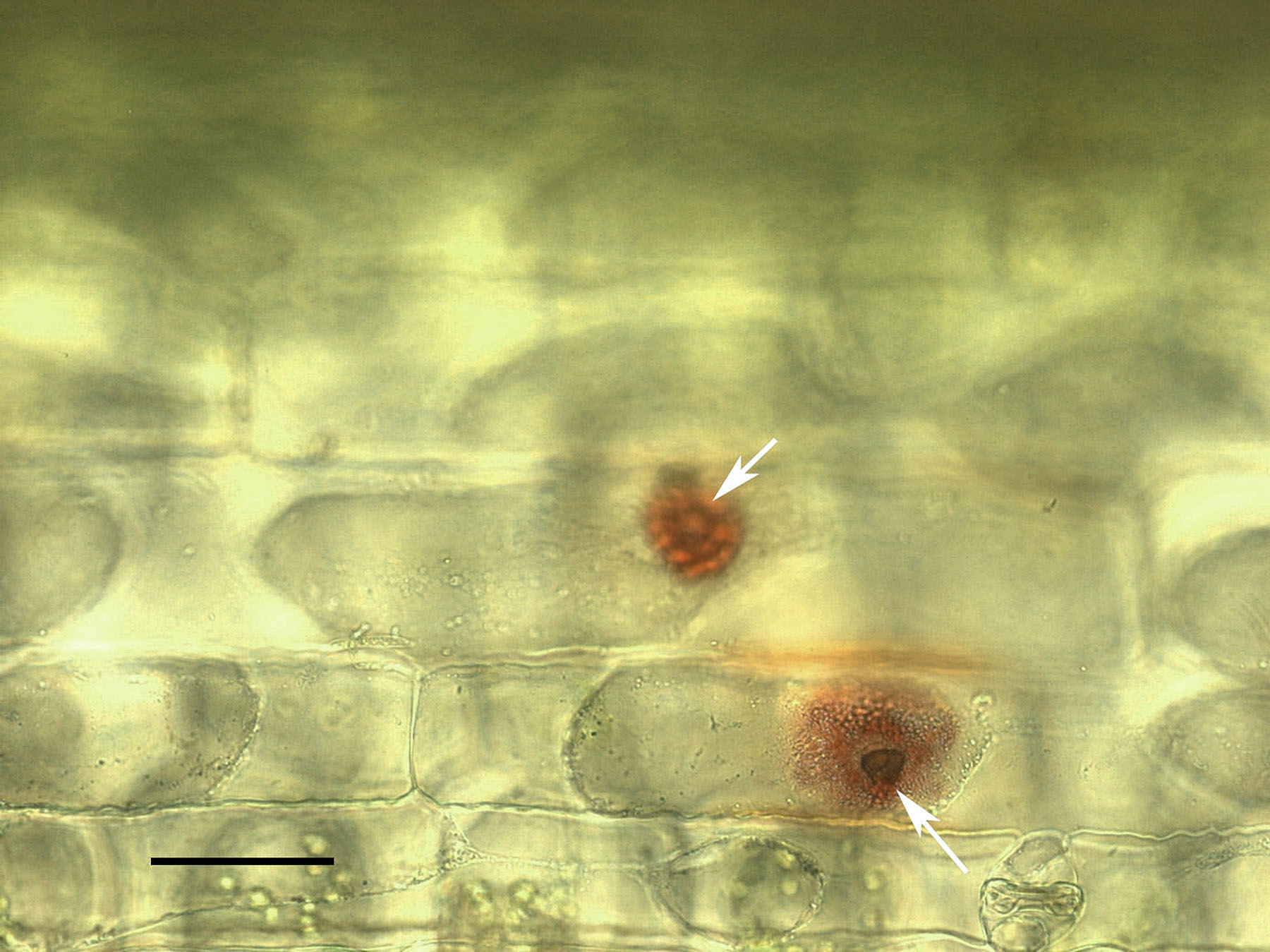

Supplement: Additional file 1: Figure S1. — M1.001 on Sugar Drip sorghum, 48 hpi, cells beneath appressoria (white arrows) plasmolyzed (result not typical). Scale bars equal to 50 μm. (JPG 302 kb) [file 12864_2016_3457_MOESM1_ESM.jpg]

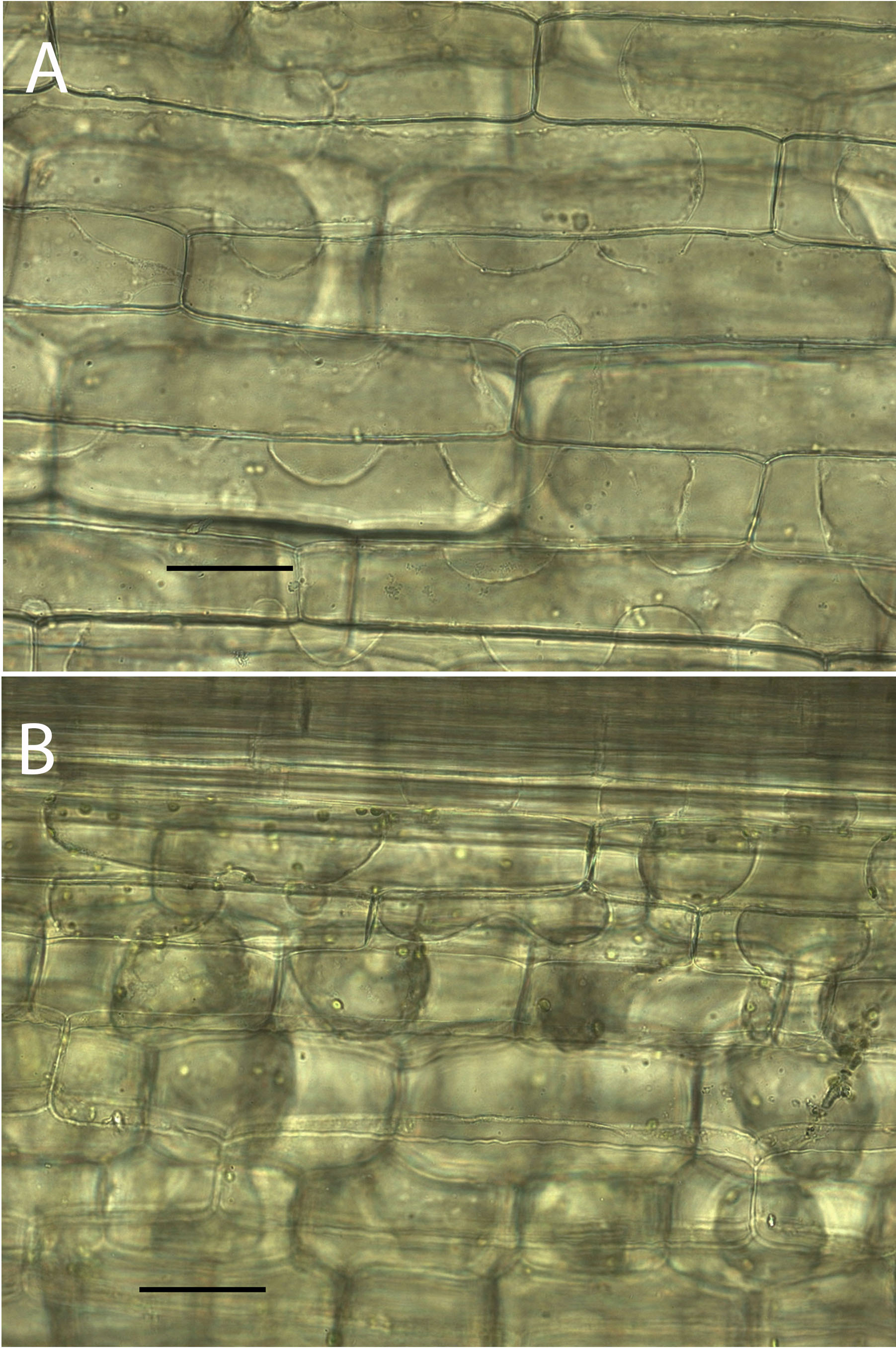

Supplement: Additional file 2: Figure S2. — Plasmolysis controls. A: Maize leaf sheath, 72 h after mock inoculation, most cells still plasmolyze. B: Sugar Drip leaf sheath, 72 h after mock inoculation, most cells still plasmolyze. Scale bars equal to 50 μm. (JPG 645 kb) [file 12864_2016_3457_MOESM2_ESM.jpg]

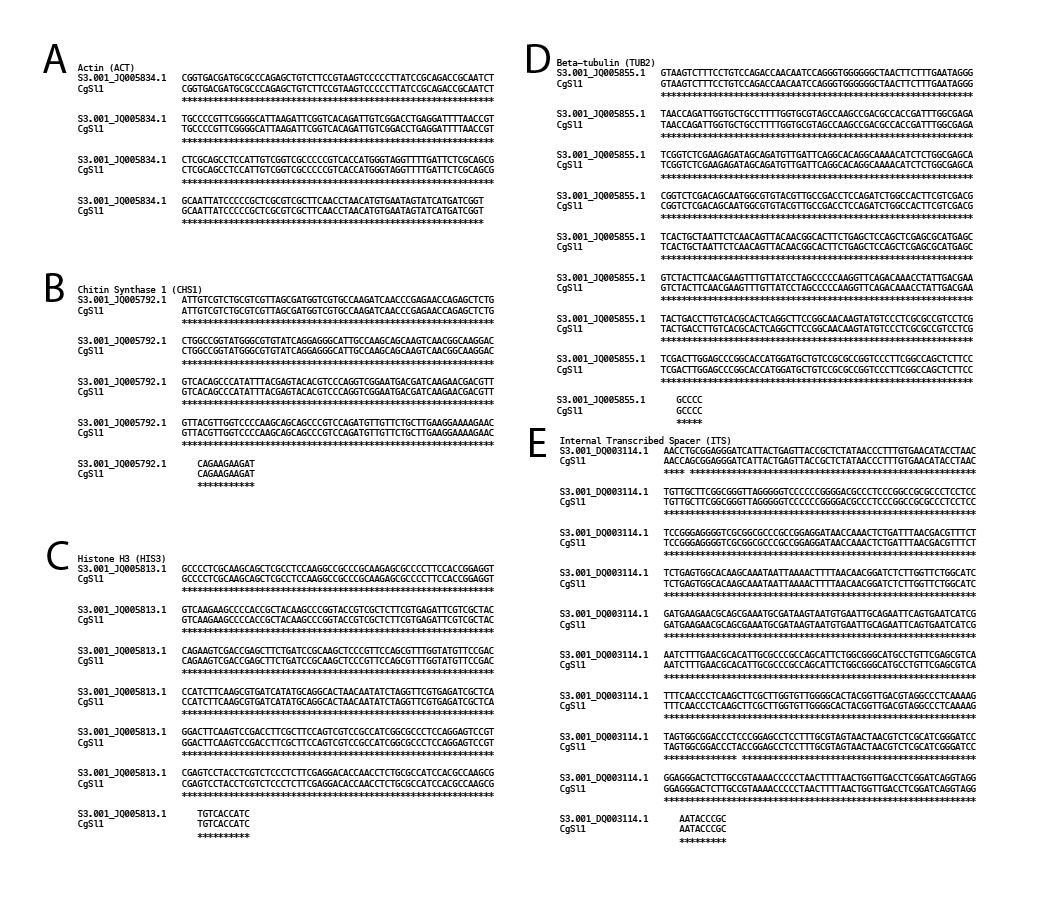

Supplement: Additional file 4: Figure S3. — Alignments of sequences of CgSl1 with species type S3.001. A: actin, B: chitin synthase, C: histone H3, D: beta-tubulin, E: ITS. Alignments done with MUSCLE version 3.7 and default parameters. (JPG 300 kb) [file 12864_2016_3457_MOESM4_ESM.jpg]
